# Targeting common denominators of DNA damage tolerance mechanisms as therapeutic strategy to cope with dynamic phenotypic changes in triple-negative breast cancer cells

**DOI:** 10.1038/s41420-026-03377-4

**Published:** 2026-10-06

**Authors:** Kathrin Niedermayer, Sara Greco, Anke Oechsle, Tanja Köhler, Klaus Pantel, Thomas W. P. Friedl, Wolfgang Janni, Brigitte Rack, Kerstin Pfister, Vanesa Gottifredi, Lisa Wiesmüller

**Affiliations:** 1 Department of Obstetrics and Gynecology, Ulm University, Ulm, Germany; 2 Institute of Tumor Biology, University Medical Centre Hamburg-Eppendorf, Hamburg, Germany; 3 Fundacion Instituto Leloir, CONICET, Buenos Aires, Argentina

**Keywords:** DNA replication, Clinical genetics, Breast cancer

## Abstract

Triple negative breast cancer (TNBC) is the most aggressive type of breast cancer with limited treatment options and a propensity towards chemoresistance. Given the lack of targetable receptors, treatment relies primarily on chemotherapy. While mechanisms leading to chemoresistance are vastly unknown, DNA damage tolerance (DDT) pathways have emerged as potential drivers of chemoresistance. Herein, we report a phenotypic characterization of four TNBC- and six non-TNBC cell lines, which supports the relevance of DDT in the TNBC response to chemotherapy. We unveiled an association between DDT pathway activation through PCNA-ubiquitination, augmented epithelial-to-mesenchymal transition (EMT) and stemness features, specifically in 3D spheroid culture from TNBC cells. Analysis of replication dynamics revealed a pronounced replication slowdown in 3D- versus 2D-cultured TNBC but not in non-TNBC cells. The exploration of DDT pathway usage in three TNBC cell lines (MDA-MB-231, Hs578t, MDA-MB-468) uncovers reliance on ZRANB3-, HLTF- and SMARCAL1-dependent fork reversal in 3D, while translesion DNA synthesis (TLS) polymerases (POLs) were more active under 2D conditions. Screening cytotoxicity with a panel of ten drugs targeting key DDT pathway components identified POLζ inhibition (JH-RE-06; IC_50_ ≤ 7 µM/48 h) and CHK1 inhibition (PF477736; IC_50_ ≤ 20 µM/48 h) as the most potent single-agent treatments in all three cell lines under both conditions. Mechanistically, both POLζ and CHK1 inhibition target several DDT pathways, in sum, causing replication slowdown, revealed consistently for CHK1. While TNBC cell lines were largely unresponsive to Olaparib mono-treatment, especially in 3D culture, all three cell lines were resensitized by combining Olaparib with low-dose POLζ-, CHK1-, or RAD51 inhibitor, except for 3D-cultured MDA-MB-231 cells, which may resist Olaparib due to their high ZRANB3 complex formation in the nucleus. These findings identify key DDT pathway components such as POLζ and CHK1 as druggable targets that alone or in combination with Olaparib may overcome chemoresistance in TNBC.

## Introduction

Breast cancer (BC) is the most common malignancy in women, characterized by a mortality/incidence ratio of around 20% in highly developed countries and around 50% in regions with medium to low development index [1, 2]. BC can be stratified into four subtypes based on the expression of certain receptors. TNBC is negative for those receptors and is the most aggressive subtype with the worst prognosis [3]. About 50% of TNBC patients develop distant metastasis, associated with a mortality rate of 75% within three months after recurrence [4, 5].

Due to its molecular heterogeneity, TNBC has limited treatment options, with chemotherapy as the main approach [5, 6]. Chemotherapeutic drugs used to combat TNBC, e.g., anthracyclines and platinum compounds, cause DNA damage, thereby challenging DNA replication and repair mechanisms [5, 7]. In case of germline *BRCA1/BRCA2* mutations or epigenetic *BRCA1* inactivation, TNBCs are susceptible to Poly(ADP-Ribose)-Polymerase inhibitor (PARPi)-treatment, an agent known to exploit homologous recombination (HR) deficiency (HRD) [8]. BRCA1 and BRCA2 also participate in DDT during DNA replication, protecting reversed forks from nucleolytic attack [9, 10]. The two major DDT pathways are TLS, in which specialized POLs, such as POLη, POLι, and POLζ, replicate across DNA lesions at the replication fork; and fork remodeling, in which specialized DNA translocases facilitate fork reversal, enabling repair of the lesion while preventing fork breakage [9, 11]. Our previous work discovered yet another DDT mechanism, a p53-POLι idling-complex mediating replication slowdown and recruitment of HLTF and ZRANB3 for safe bypass of replication barriers via fork reversal [12–15]. RAD51, commonly known for its role in HR, and PARP1 are also involved in the formation and protection of reversed forks [9, 10]. A more recently discovered DDT mechanism involves PRIMPOL-mediated repriming behind stalled forks, resulting in ssDNA gap accumulation and fill-in by POLζ or sealing by RAD51-mediated template switching [9, 10, 16–18].

TNBC cells frequently undergo EMT, which has been linked with changes in DNA damage responses (DDRs) and DDT mechanisms [3, 19, 20]. Given the involvement of DDT mechanisms in drug resistance [21, 22], small-molecule inhibitors have gained interest. Recent strategies aim at inhibitors of fork remodeling or repriming [7, 23, 24], but overall, the TLS pathway has been the primary focus [25–27]. TLS inhibitors were proposed as a novel class of adjuvant anti-cancer agents, enhancing cytotoxicity by preventing cancer cell recovery after DNA damage [27, 28].

In this work, we screened ten BC cell lines in 2D-monolayer and 3D-spheroid cultures and detected augmented EMT, 3D-stemness, and 3D-replication slow-down in TNBC but not in non-TNBC cell lines. Targeting key DDT pathway components by knockdown or pharmacological inhibition revealed differential DDT pathway usage of TNBC cells in 2D and 3D and identified promising drug targets for (re-)sensitization of TNBC cells regardless of culture conditions and the plasticity of the replication phenotype.

## Results

### TNBC cell lines show elevated EMT and stemness features

**Fig. 1:**
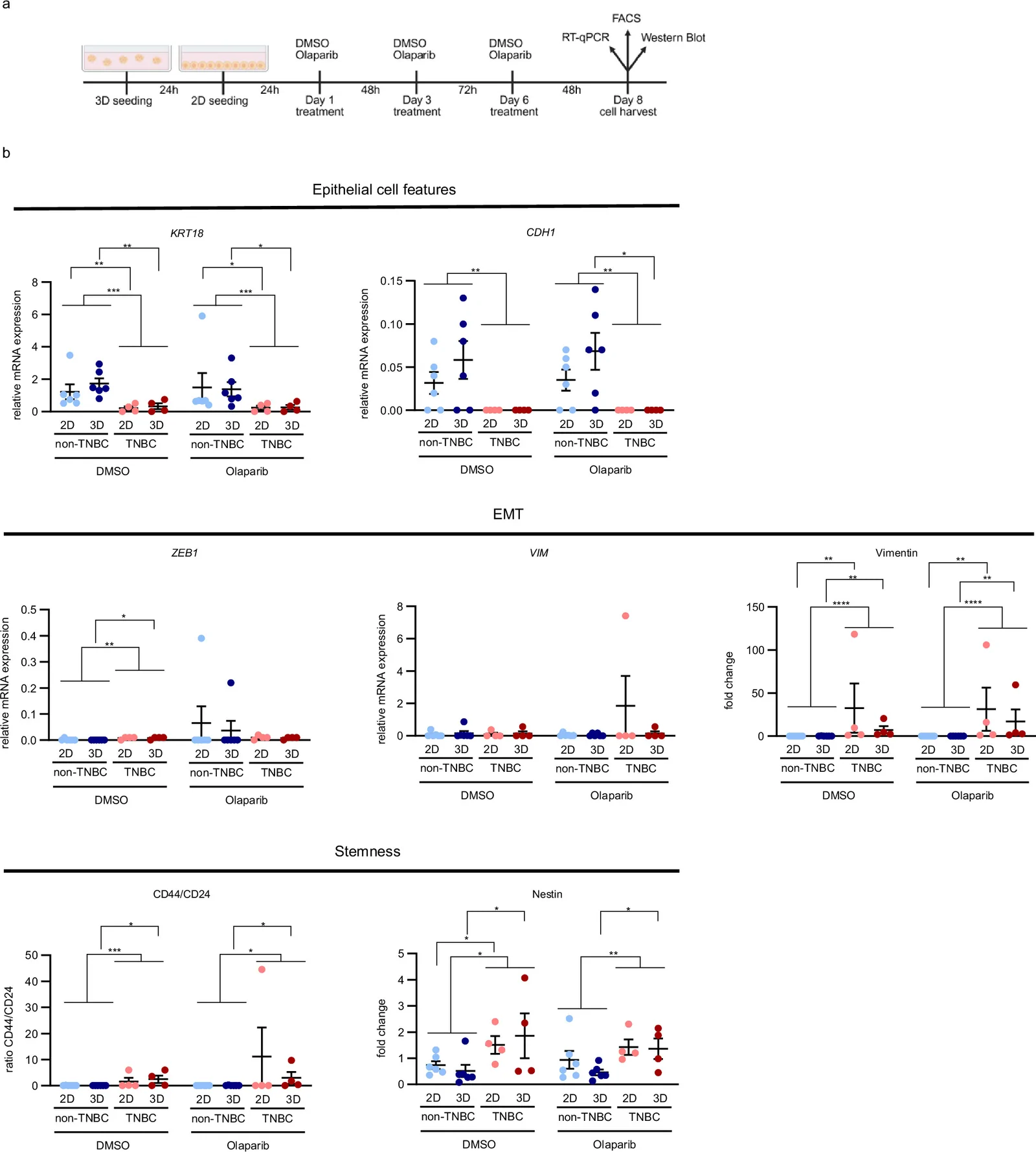
Analysis of epithelial cell, EMT and stemness features of non-TNBC and TNBC cell lines with and without Olaparib treatment. **a** Scheme of experimental procedure. Cells were treated for eight days before RT-qPCR, western blot, and FACS analysis, with Olaparib (1 µM) and mock-treatment (DMSO) being administered every 2–3 days. **b** Phenotypes after DMSO and Olaparib treatment. Upper panels with epithelial cell features: Relative mRNA expression levels of *KRT18* and *CDH1* are shown. Middle panels with EMT features: Relative mRNA expression levels of *ZEB1* and *VIM*, and relative protein levels of Vimentin. mRNA expression levels were determined by RT-qPCR, normalized to at least two of three housekeeping genes (*ACTB*, *RPL4*, *PPIA*) with the 2^-∆Ct^ method. Lower panels with stemness features: Ratio of CD44/CD24 and relative protein levels of Nestin are shown. Western blot analyses of Vimentin and Nestin, normalized to GAPDH and internal standard MDA-MB-468 sample (2D, untreated) from the same blot are presented. Mean fluorescent intensities of CD44 and CD24 were measured by FACS in the live cell gate in respective samples. Dots show the mean values from two to three independent experiments (*N* = 2–3), with each dot representing a single cell line. Blue dots for non-TNBC, red for TNBC, with light blue and light red for 2D-, and dark blue and dark red for 3D-cultivation. Non-TNBC: MDA-MB-453, BT474, T47D, MCF-7, MDA-MB-134-VI, CTC-ITB-01. TNBC: MDA-MB-231, Hs578t, MDA-MB-468 and MDA-MB-436. Bars and whiskers indicate mean values ± SEM of the means for different cell lines. Significances were calculated by Mann–Whitney *U* test, in case of statistical significance being reached with Kruskal–Wallis *H*-test, using GraphPad Prism 9. *p < 0.05; **p < 0.01; ***p < 0.001; ****p < 0.0001.

Previous work revealed differential use of DDT mechanisms during DNA replication in human embryonal and adult stem (cell-like) cells (SCs) as compared to their differentiated counterparts [13]. To understand whether differences in stemness of BC cells will also impact DDT pathway choices and thereby chemoresistance, we analyzed TNBC (MDA-MB-231, Hs578t, MDA-MB-468, MDA-MB-436) and non-TNBC cell lines (MDA-MB-453, BT474, T47D, MCF-7, MDA-MB-134-VI, CTC-ITB-01, see Supplementary Table 1) cultivated either in 2D-monolayers or 3D-spheroid cultures. First, we investigated if these BC subtypes show differences in EMT and/or related stemness features [29] and if differences depend on the cultivation method and the levels of DNA damage (untreated versus clinically relevant genotoxic drugs, i.e., Olaparib, Carboplatin, or Doxorubicin; Fig. 1a; Supplementary Fig. 1a).

We first determined mRNA expression levels of markers of epithelial cells, i.e., *KRT18* and *CDH1* [30], versus mesenchymal cells, i.e., *ZEB1* [31] and *VIM* [32]. *KRT18* and *CDH1* showed higher levels in non-TNBC lines, mostly independently of treatment and cultivation methods. *ZEB1* expression rose in TNBC lines when exposed to DMSO or Carboplatin. No differences in *VIM* mRNA expression were found (Fig. 1b, Supplementary Fig. 1a), but western blot analysis revealed higher Vimentin protein levels in TNBC cells, which in 3D was augmented by Carboplatin-treatment (Fig. 1b, Supplementary Fig. 1a, b). To pinpoint stemness, we determined the ratio of CD44/CD24 signals by FACS analysis [33] and protein expression of Nestin, which reveals poor prognosis [34]. Both stemness markers consistently increased in the TNBC versus non-TNBC groups, particularly when focusing on 3D-cultured cells (Fig. 1b, Supplementary Fig. 1a, b). Doxorubicin and Carboplatin treatments augmented CD44/CD24 signals also in 3D-cultured non-TNBC cells (Supplementary Fig. 1a). These findings indicate that the TN feature and also genotoxic stress augmented EMT and stemness in BC, particularly in 3D.

### TNBC cell lines decelerate replication in 3D

**Fig. 2:**
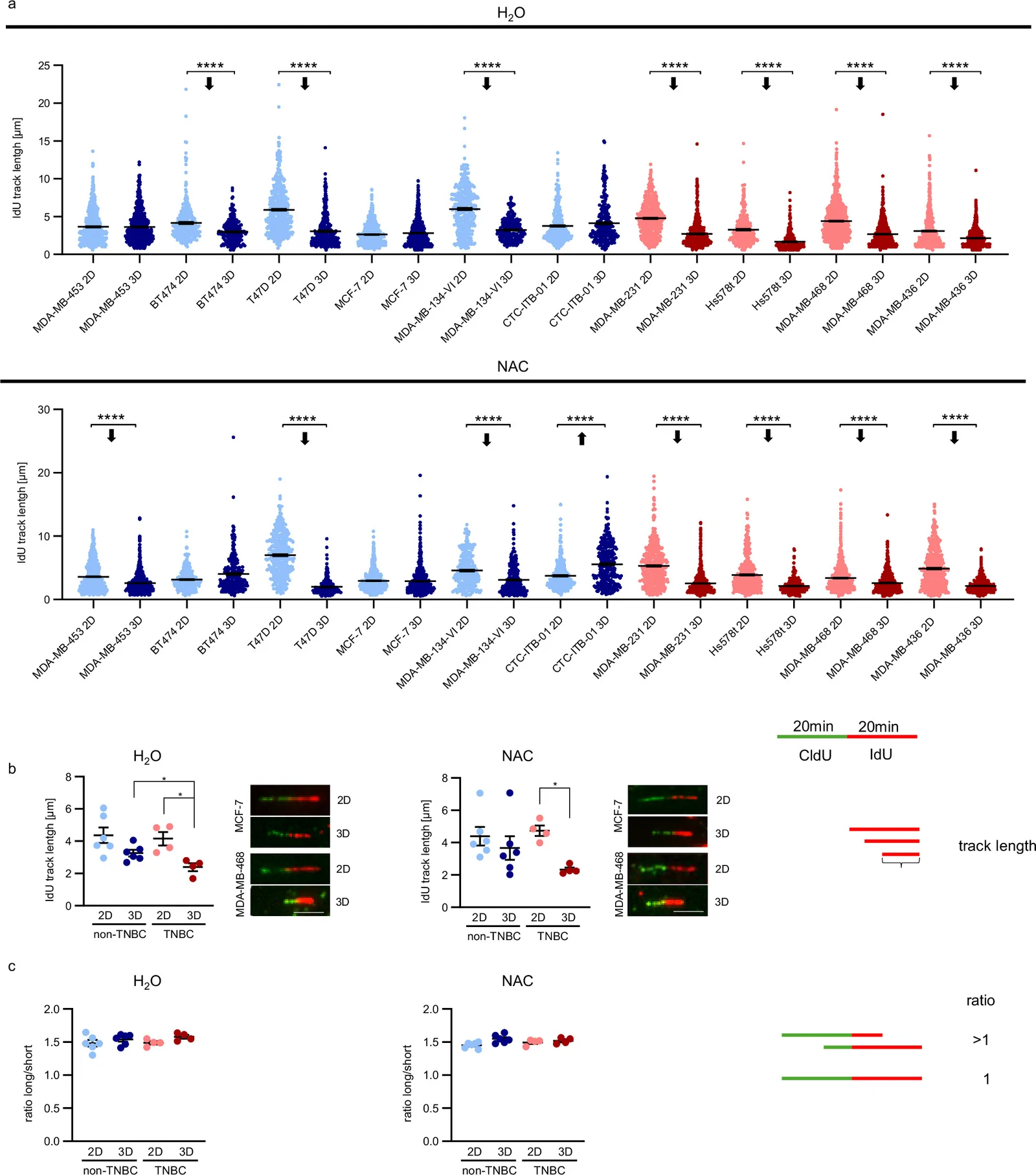
DNA replication dynamics in non-TNBC and TNBC cell lines using the DNA fiber spreading assay. **a** DNA replication elongation. IdU-labeled track lengths in µm (mean ± SEM of ≥243 fibers from two individual experiments) are shown in blue for non-TNBC cell lines (MDA-MB-453, BT474, T47D, MCF-7, MDA-MB-134-VI and CTC-ITB-01) and in red for TNBC cell lines (MDA-MB-231, Hs578t, MDA-MB-468 and MDA-MB-436). Light blue and light red for 2D, and dark blue and dark red for 3D cultivation, treated with H_2_O or NAC, respectively. Significances were calculated by Mann–Whitney *U* test, in case of statistical significance being reached with Kruskal–Wallis *H*-test, using GraphPad Prism 9. ****P < 0.0001. Black arrows indicate the direction of change in track length. **b** Comparison of IdU track lengths of non-TNBC and TNBC cell lines in 2D and 3D treated with H_2_O or NAC, respectively. Representative fiber images are shown for MCF-7 and MDA-MB-468 in 2D and 3D. Scale bar equals 5 µm. **c** Replication asymmetry. Comparison of ratios of long/short fiber tracks (CldU or IdU) of non-TNBC and TNBC cell lines in 2D and 3D treated with H_2_O or NAC. Dots show the mean values from two independent experiments (*N* = 2), with each dot representing a single cell line. Bars and whiskers indicate mean values ± SEM of the means for different cell lines. Cell lines used and calculation of significances were as in (**a**). *P < 0.05.

To analyze replication dynamics as a function of stemness, we performed DNA fiber spreading assays in both groups of BC lines in 2D and 3D. Measurements of IdU-labeled replication track lengths revealed no or inconsistent changes in track lengths from 2D to 3D cultures for the six non-TNBC lines, but extremely consistent shortening from 2D to 3D in all four TNBC lines. This was true both before and after inclusion of NAC (N-Acetylcysteine) [35], a scavenger of reactive oxygen species (Fig. 2a). These observations were also evidenced in group comparisons, confirming significantly shorter tracks in TNBC lines cultured in 3D versus 2D (Fig. 2b). To test if replication fork stalling was the underlying reason for shorter tracks, we calculated the ratios of long/short tracks in bi-colored fibers [36]. The lack of any significant differences (Fig. 2c) suggests that the consistent downregulation of replication speed in 3D-spheroids observed in TNBC lines was not due to replication fork stalling.

### DDT activation by PCNA-ubiquitination in TNBC and recruitment of translocase ZRANB3 in 3D

**Fig. 3:**
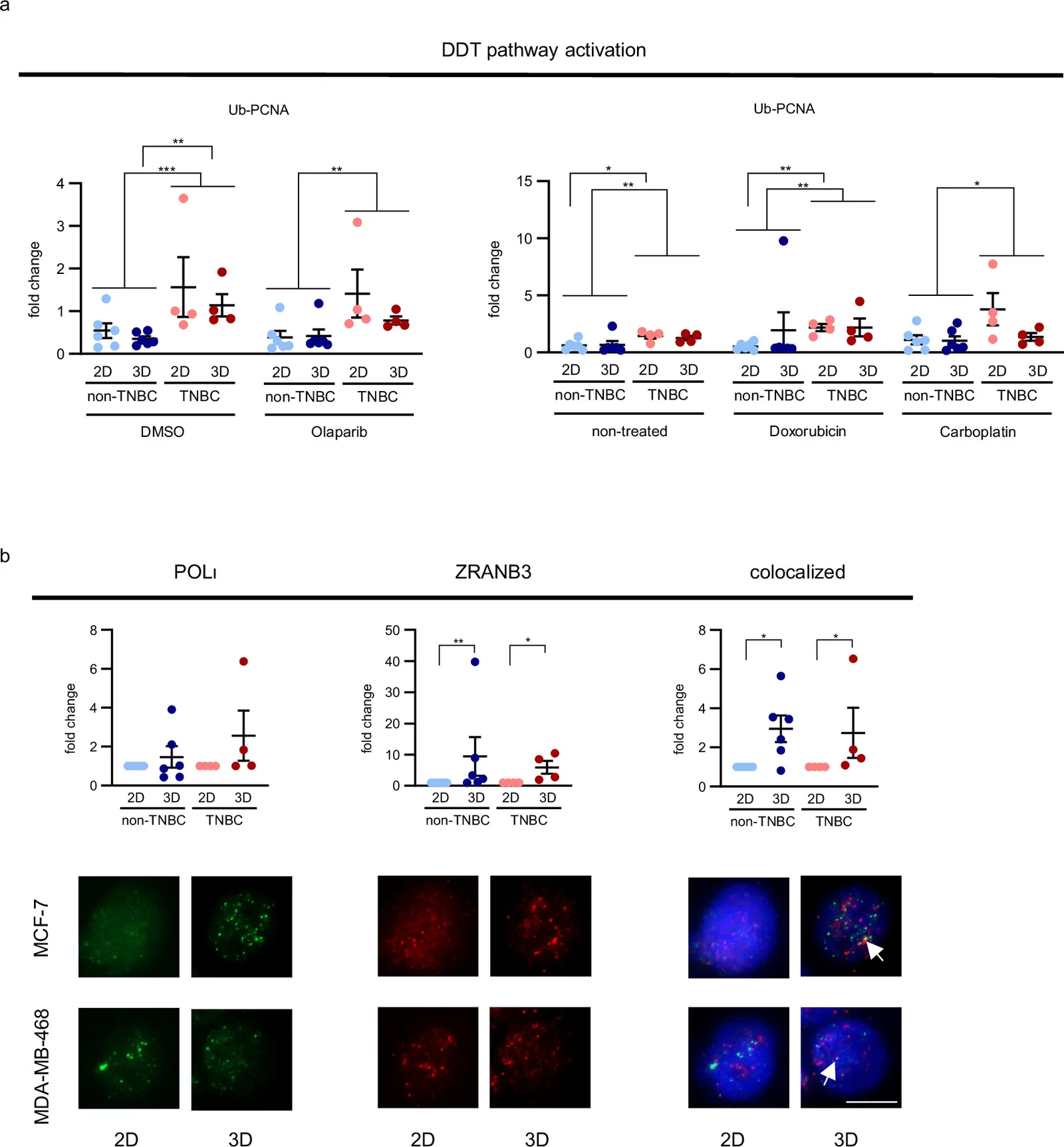
DDT pathway activation in non-TNBC versus TNBC cells. **a** PCNA-ubiquitination. Comparison of the relative protein levels of Ub-PCNA of non-TNBC and TNBC cell lines treated with DMSO and Olaparib in the left panel and with H_2_O (non-treated), Doxorubicin (0.31 µM), and Carboplatin (10 µg/ml) in the right panel. The protein levels were determined by western blot analysis and normalized to GAPDH and the internal standard MDA-MB-468 sample (2D, non-treated). Representative western blots are shown for MCF-7 and MDA-MB-468 in Supplementary Fig. 2c. Bars and whiskers indicate mean values ± SEM of the means for different cell lines. Significances were calculated by Mann–Whitney *U* test, in case of statistical significance being reached with Kruskal–Wallis *H*-test, using GraphPad Prism 9. *N* = 2; *P < 0.05; **P < 0.01; ***P < 0.001. **b** Nuclear foci formation of DDT pathway components. Fold changes of POLι, ZRANB3, and colocalized foci in non-TNBC and TNBC cell lines. For the fold change, the mean value for each cell line in 3D was normalized to the mean value for the respective 2D sample in two individual experiments (absolute mean values in 2D for POLι, ZRANB3 and colocalized are for MDA-MB-453: 16.02, 5.98, 0.02; BT474: 71.84, 7.34, 0.04; T47D: 64.26, 22.52, 0.03; MCF-7: 18.41, 5.09, 0.01; MDA-MB-134-VI: 34.10, 9.87, 0.04; CTC-ITB-01: 37.21, 13.21, 0.57; MDA-MB-468: 10.88, 2.95, 0.02; MDA-MB-231: 37.78, 25.37, 0.03; Hs578t: 7.79, 12.32, 0.02; MDA-MB-436: 5.52 13.06, 0.01). Non-TNBC (blue) and TNBC (red) cell lines, with light blue and light red for 2D, and dark blue and dark red for 3D cultivation, respectively. Bars and whiskers indicate mean values ± SEM of the means for different cell lines. Significances were calculated as in (**a**). *P < 0.05; **P < 0.01. Representative images are shown for MCF-7 and MDA-MB-468, white arrows indicate colocalization, scale equals 10 µm.

The observed slowdown of replication in 3D-cultured TNBC cells guided us to investigate a key feature of DDT activation, namely ubiquitination of PCNA, reflecting perturbations at the replication fork [37]. Here, we determined the level of monoubiquitinated PCNA (Ub-PCNA) by western blot analysis (Fig. 3a). Higher Ub-PCNA levels were seen in TNBC lines across all treatments and culture conditions (Fig. 3a), while no differences in PCNA expression were detectable (Supplementary Fig. 2a, c). Although most non-TNBC and all TNBC lines harbor mutations in *TP53* (Supplementary Table 1), we studied the formation of nuclear POLι foci, which in wild-type *TP53* cells was previously shown to correlate with activation of ZRANB3-mediated fork reversal [12]. Neither nuclear POLɩ foci numbers nor POLɩ protein levels showed significant differences (Fig. 3b, Supplementary Fig. 2b, c). Our preceding study identified increased formation of POLι-ZRANB3 complexes with prolonged residence times at the fork as a discriminatory molecular feature of DNA replication in SCs [13]. Here, immunofluorescence microscopic analysis of nuclear ZRANB3 foci numbers showed an increase in 3D versus 2D in both non-TNBC and TNBC cells, and colocalizing foci of POLɩ and ZRANB3 followed a similar pattern (Fig. 3b). In conclusion, replication perturbations labeled by Ub-PCNA are more frequent in TNBC, both in 2D and 3D conditions, whereby ZRANB3 foci accumulate more frequently in 3D. Hence, only in 3D-cultured TNBC cells do we see the combination of PCNA ubiquitination and ZRANB3 recruitment, which associates with track length shortening.

### Dissecting DDT mechanisms through knockdown or inhibition of key components reveals a preference for fork reversal in 3D-cultured TNBC cells

**Fig. 4:**
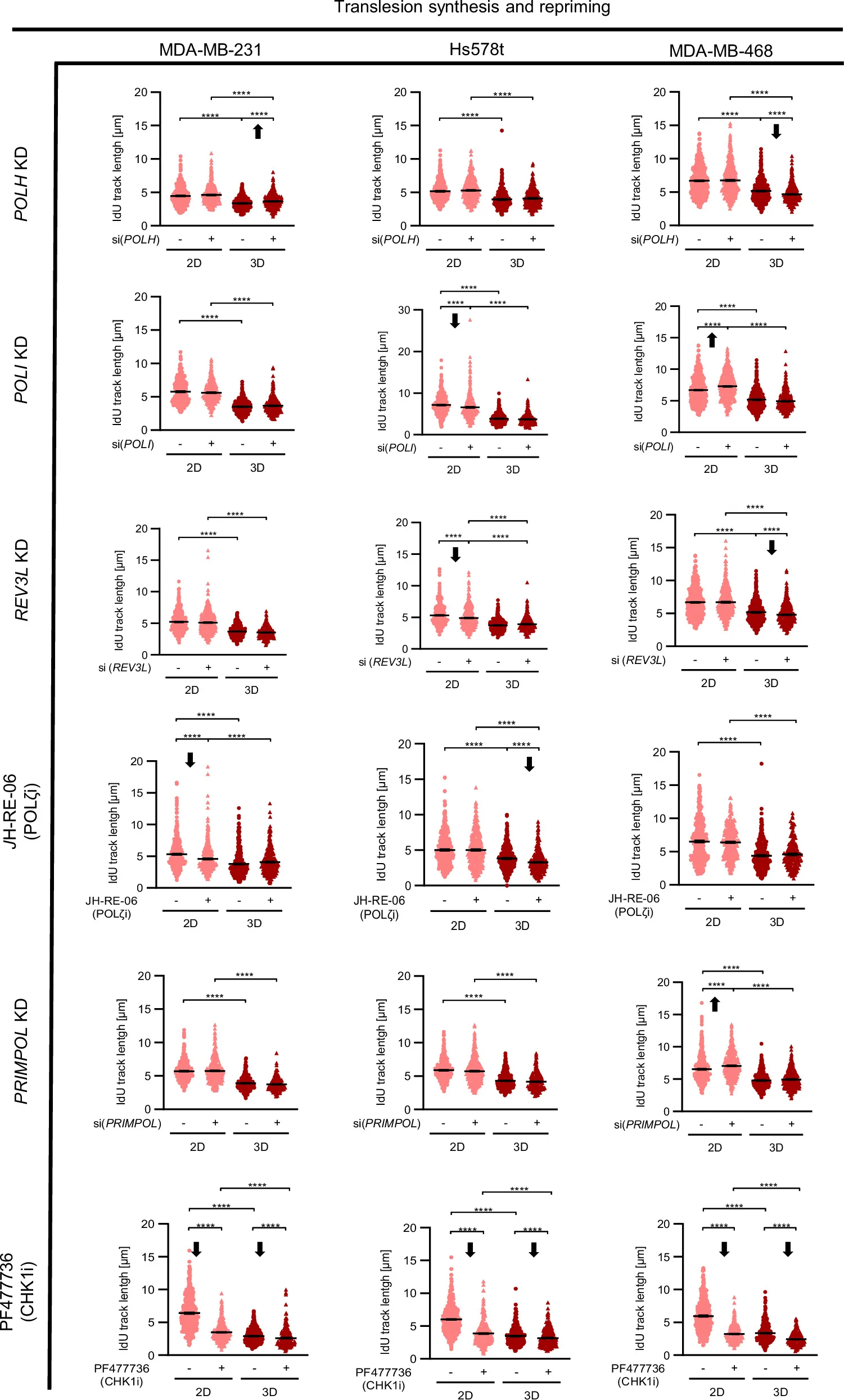
Targeting translesion synthesis and repriming in TNBC cells. Comparison of IdU-labeled track lengths in µm (Mean ± SEM from ≥375 fibers from three to four individual experiments) in MDA-MB-231, Hs578t, and MDA-MB-468 cells in 2D (light red) and 3D (dark red) after knockdown of *POLH*, *POLI*, *REV3L*, and *PRIMPOL* (10 nM siRNA each) or inhibition of POLζ with JH-RE-06 and CHK1 with PF477736 using the respective IC_50_ concentration (Table 2). Significances were calculated by Mann–Whitney *U* test, in case of statistical significance being reached with Kruskal–Wallis *H*-test, using GraphPad Prism 9. Mean ± SEM; ****P < 0.0001. Black arrows indicate the direction of change in track length after knockdown.

**Fig. 5:**
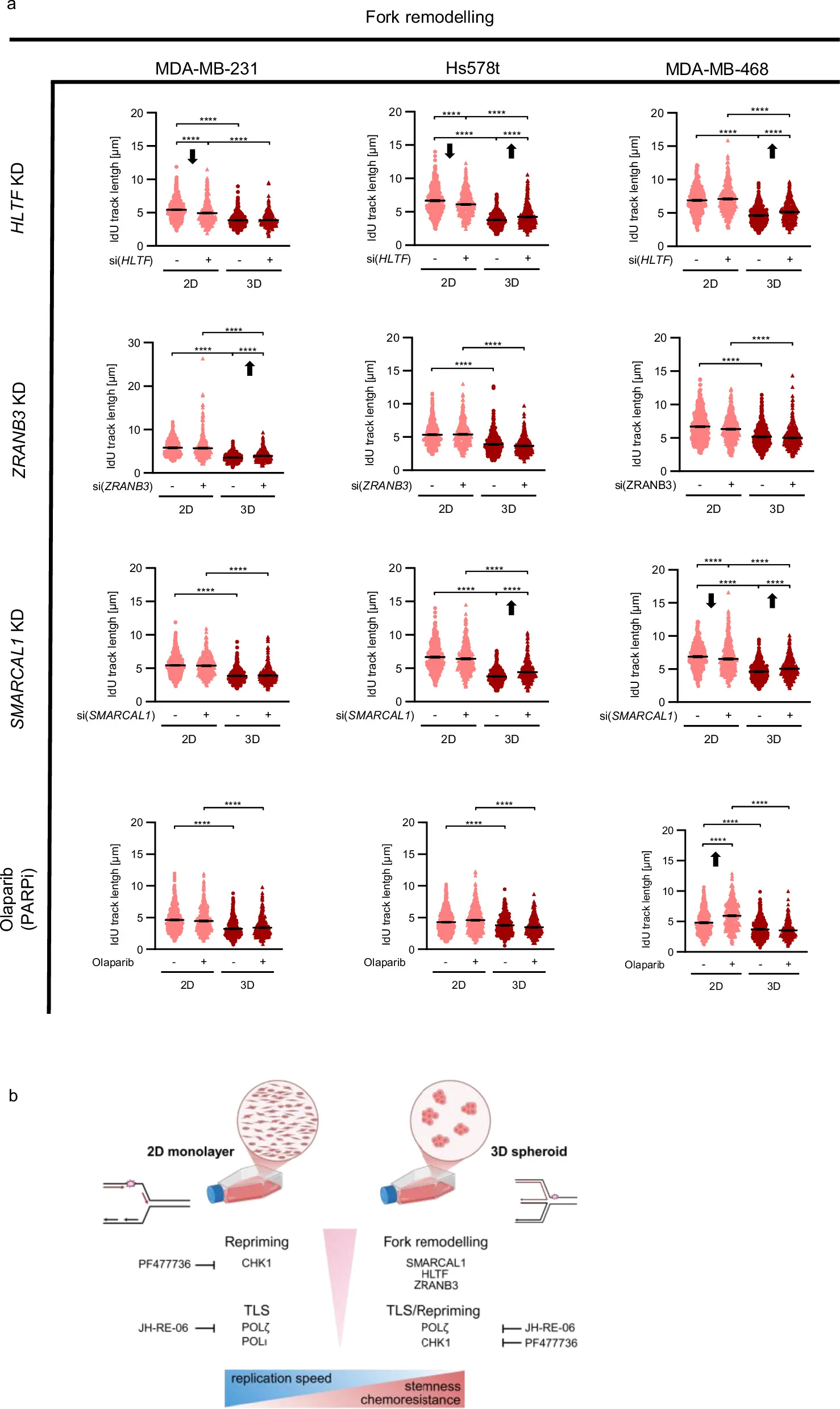
Targeting fork remodeling in TNBC cells. **a** Comparison of IdU-labeled track lengths in µm (Mean ± SEM from ≥516 fibers from three to four individual experiments) in MDA-MB-231, Hs578t and MDA-MB-468 cells in 2D (light red) and 3D (dark red) after knockdown of *HLTF*, *ZRANB3* and *SMARCAL1* (10 nM siRNA each) or inhibition of PARP1 with Olaparib (10 µM). Significances were calculated by Mann–Whitney *U* test, in case of statistical significance being reached with Kruskal–Wallis *H*-test, using GraphPad Prism 9. Mean ± SEM; ****P < 0.0001. Black arrows indicate the direction of change in track length after knockdown. **b** Schematic summary. TNBC cells cultivated in 3D-spheroids augment stemness features and slow down DNA replication as compared to 2D-monolayers. This change in DNA replication dynamics relies on differential DDT pathway usage: In 2D TNBC cells, they preferentially use repriming activated by CHK1 and TLS executed by POLζ and POLɩ. In 3D, additional use of fork remodeling by SMARCAL1, HLTF, and/or ZRANB3 decelerates the fork and conveys chemoresistance. Screening different druggable DDT pathway components identifies CHK1 (PF477736) and POLζ (JH-RE-06) as common targets for potential use in mono- and/or combination therapies against TNBC.

To gain deeper insight into the mechanisms triggering track length differences in TNBC cells (3D versus 2D), we performed knockdown experiments for several DDT pathway players, followed by DNA fiber spreading analysis in TNBC lines MDA-MB-231, Hs578t, and MDA-MB-468 in 2D and 3D (Figs. 4, 5; Supplementary Figs. 3–5). To test the hypothesis of differential involvement of TLS, POLη, POLι, and POLζ were targeted in addition to PRIMPOL, which is engaged in repriming [11, 38–40]. The alternative DDT pathway to TLS and repriming is fork reversal; therefore, HLTF, ZRANB3, and SMARCAL1 were downregulated [9, 41].

Shorter tracks were observed after *POLH* knockdown in 3D-cultured MDA-MB-468 and longer tracks in MDA-MB-231 (Fig. 4), which can be explained by canonical and non-canonical roles of POLη [42] in TLS and D-loop extension during replication-associated recombination, respectively. Knockdown of *POLI* led to opposing phenotypes in 2D-cultured Hs578t versus MDA-MB-468 cells (Fig. 4), which can be explained by a context-dependent involvement of POLι in TLS or fork remodeling [13, 15]. Consistent with an exclusive role in TLS, downregulation of REV3L, the catalytic subunit of POLζ, shortened tracks, namely in 2D-cultured Hs578t and 3D-cultured MDA-MB-468 cells. After *PRIMPOL* knockdown, unexpectedly lengthening of tracks in 2D-cultured MDA-MB-468 cells was the only significant change observed (Fig. 4). Deceleration of replication by PRIMPOL, seen here rather than repriming-induced acceleration, may relate to its antagonistic roles towards TLS [15, 40, 43].

Knockdown of HLTF resulted in track length decreases in 2D-cultured MDA-MB-231 and Hs578t cells but increases in 3D-cultured Hs578t and MDA-MB-468 cells (Fig. 5a). Such bimodal HLTF effects are in line with its roles in mediating both mono- and polyubiquitination of PCNA towards TLS and fork reversal, respectively [44, 45]. Knockdown of translocases ZRANB3 and SMARCAL1 primarily resulted in track lengthening in 3D-cultured cells, namely after *ZRANB3* knockdown in MDA-MB-231 and after *SMARCAL1* knockdown in Hs578t as well as MDA-MB-468 cells (Fig. 5a). To test fork stalling, we determined the ratio of long/short tracks. Only MDA-MB-231 showed a significant increase after *ZRANB3* knockdown in 2D, suggesting that track shortenings were not caused by stalling (Supplementary Fig. 4). These results, summarized in Table 1, highlight the heterogenous nature of TNBC lines and indicate that TLS and fork reversal are used in TNBC in a cell type- and condition-dependent manner. Under 3D-conditions, a preference for fork reversal becomes evident, though dependence on individual fork reversal factors, i.e., HLTF, ZRANB3, or SMARCAL1, remains cell type-specific.

**Table 1:**
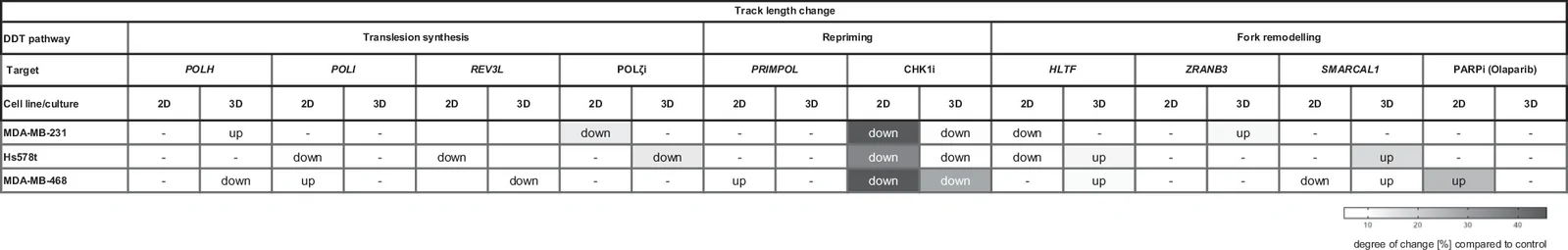
Impact of knockdown and inhibition of DDT pathway components.

Summary of DNA fiber assay results after knockdown or inhibition (i) of key DDT components (see Figs. 4 and 5). Significant changes in IdU track length after knockdown of *POLH, POLI, REV3L, PRIMPOL*, *HLTF, ZRANB3* and *SMARCAL1*, and inhibition of POLζ, CHK1, and PARP1 are marked. A track length increase is indicated as “up” and a track length decrease is indicated as “down”. The heat map illustrates the degree of change in track length, with dark grey indicating the highest degree and white the lowest. Every value above the median was colored grey, everything below the median was colored white, and the median was set to white.

### TNBC cell lines show high sensitivity to POLζ and CHK1 inhibition in 2D- and 3D-culture

Next, we analyzed sensitivities of TNBC lines to inhibition of key DDT pathway and related DDR components. As shown in Supplementary Fig. 6 and compiled in Table 2, IC_50_-values were determined via MTT assay after treatment of MDA-MB-231, Hs578t and MDA-MB-468 cells in 2D- and 3D-cultures for 48 h with inhibitors of (i) TLS-POLs: POLηi (PNR-7-02), POLιi (MK-886), POLζi (JH-RE-06); (ii) repriming activation: CHK1i (PF477736), (iii) fork remodeling: RAD51i (B02), MRE11i (Mirin), DNA2i (C5), ATRi (AZD-6738); (iv) break-induced replication (BIR): RAD52i (L-Dopa) and origin firing: CDC7i (XL413) [9, 46–48].

**Table 2:**
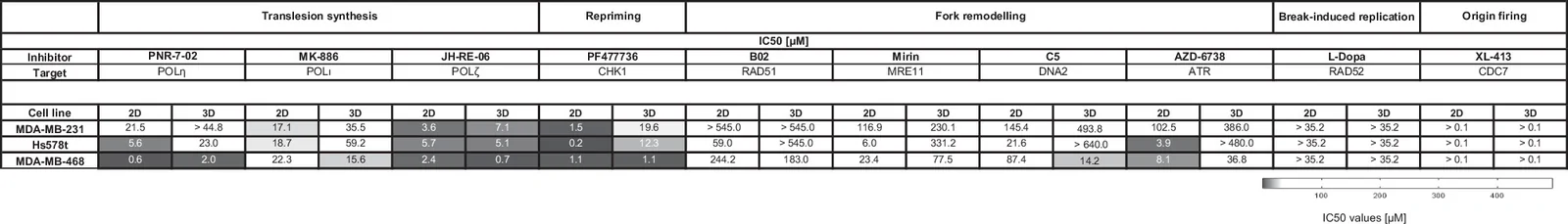
Sensitivity of TNBC cell lines MDA-MB-231, Hs578t and MDA-MB-468 to different DDT pathway inhibitors.

TNBC cell lines MDA-MB-231, Hs578t, and MDA-MB-468 were treated with different concentrations of PNR-7-02, MK-886, JH-RE-06, PF477736, B02, Mirin, C5, AZD-6738, L-Dopa, and XL-413 targeting components of DDT pathways as indicated. Cell viability was determined by MTT assay in 2D and 3D cultivation. IC_50_-values (µM) were determined 48 h post-treatment from combined viability data of three independent experiments performed in duplicates each (see viability curves in Supplementary Fig. 6). The heat-map visualizes differences in IC_50_-values, with grey indicating lower IC_50_-values (higher sensitivity) and white indicating higher IC_50_-values (lower sensitivity). Each value below the median was colored dark grey, each value above the median was colored white, and the median was set to white. Statistically significant differences between IC_50-_values were determined and are summarized in Supplementary Table 2. Values marked with “>“ indicate that the IC_50_ exceeded the highest tested concentration and was therefore not calculated.

The heat map in Table 2 unveils common patterns of responses to drug treatments despite heterogeneities across the three TNBC lines and culture conditions. The most pronounced effects were observed with inhibitors targeting TLS (POLζi > POLηi > POLιi) and repriming (CHK1i), whereby the lowest IC_50_-values were measured for POLζi (≤7 µM) and CHK1i (≤20 µM) in all three cell lines under both conditions. Some agents caused cell line-specific effects (e.g., ATRi in 2D) (Table 2), and most drugs caused differences between 2D- and 3D-values in at least two cell lines (Supplementary Table 2). Strikingly, RAD52i and CDC7i treatments showed no impact on viabilities, and RAD51i caused only moderate effects (Table 2).

Next, we performed DNA fiber spreading assays to analyze the impact of POLζ and CHK1 inhibition on DNA replication dynamics in all three cell lines in 2D and 3D. Additionally, we inhibited PARP1 with the clinically approved drug Olaparib [8], as it was reported to antagonize RECQ1-mediated restart of reversed forks [9]. Strikingly, incubation with CHK1i slowed down replication in all cell lines regardless of the cultivation method, thereby almost neutralizing the track length differences between 2D and 3D (Fig. 4). POLζi decelerated replication, namely in 2D-cultured MDA-MB-231 and 3D-cultured Hs578t cells (Fig. 4), while PARPi Olaparib accelerated replication in 2D-cultured MDA-MB-468 cells (Fig. 5a). Replication fork stalling was excluded as reason for track shortenings, because long/short track ratios did not increase (Supplementary Fig. 4). A summary of all fiber track length results can be found in Table 1, suggesting that in all cell lines CHK1 is crucial for fast replication in 2D with contributions by POLζ- and POLɩ-dependent TLS in MDA-MB-231 and Hs578t cells, respectively. Taken together, this data identifies POLζi and CHK1i, both affecting fork elongation, as drugs that sensitize TNBC lines in 2D- and 3D-culture.

### TNBC cell lines can be resensitized to Olaparib by combined treatment with DDT pathway inhibitors

To identify potential combination strategies to resensitize TNBC cells to therapy, we first analyzed the efficacy of clinically used chemotherapeutic agents [8, 49, 50] in the three TNBC lines. Therefore, the MTT viability assay was performed with Olaparib, Doxorubicin, and Carboplatin during 2D- and 3D-cultivation (Supplementary Table 3). In all three TNBC lines, Olaparib showed moderate effects with high IC50-values in 2D and resistance to treatment in 3D. Chemoresistance was also observed in Carboplatin-treated MDA-MB-231 and Hs578t cells, while MDA-MB-468 responded in both culture conditions with IC50 values ≤ 15 µM. Treatment with Doxorubicin severely reduced viabilities in all three cell lines (IC50 values ≤ 50 µM), with significantly lower IC_50_-values in 2D compared to 3D.

Due to the weak response to the clinically approved drug Olaparib, particularly in 3D, we studied combination treatments with the most interesting DDT pathway inhibitors identified in single treatments (Table 2). Thus, we combined POLζi or CHK1i, both at the IC20, with Olaparib at different concentrations (Table 3). In the same way, we combined RAD51i with Olaparib, aiming at concomitant inhibition of fork remodeling and restart of reversed forks, respectively [9, 46]. Finally, we added the efflux pump inhibitor (P-Glycoprotein/MDR1) Verapamil [51] to take multidrug resistance (MDR) into account. However, Verapamil did not significantly reduce IC50-values compared to Olaparib monotreatment in any of the TNBC lines (Table 3). All three cell lines were sensitized in 2D by the combination of Olaparib with POLζi, CHK1i, or RAD51i (Table 3). In 3D combined effects were seen in Hs578t and in MDA-MB-468 but not MDA-MB-231 (Table 3). Our data suggest that resistance to Olaparib can be overcome by a combination with DDT pathway inhibitors JHRE06 (POLζi), PF477736 (CHK1i), and even B02 (RAD51i), whereby B02, like Olaparib, showed a limited effect in monotreatment (Table 2), revealing synthetic lethality.

**Table 3:**
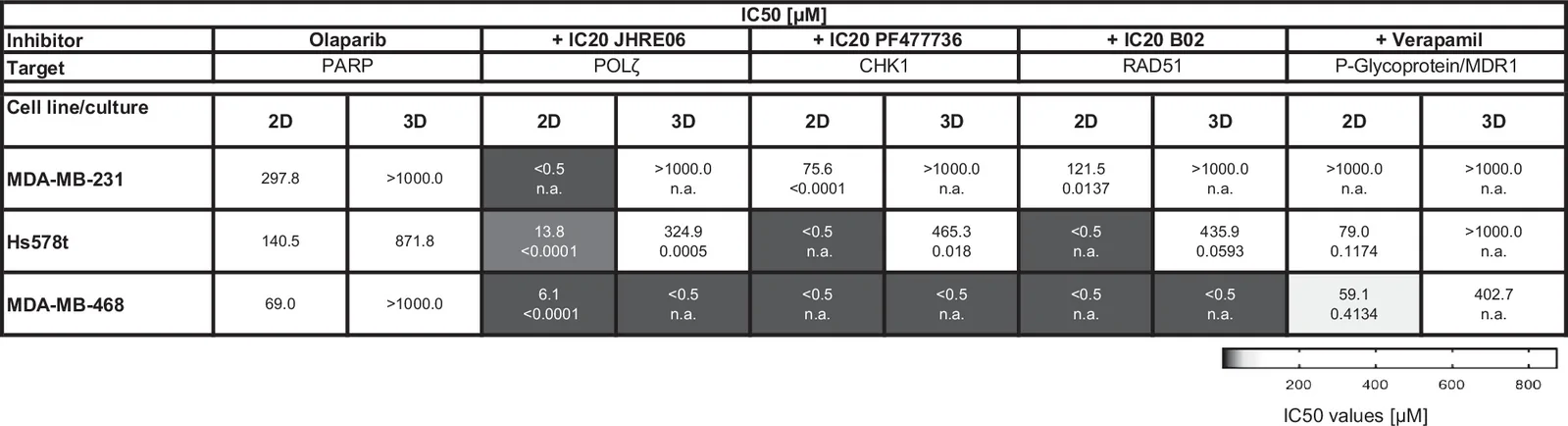
Sensitivity of TNBC cell lines to combined treatment with Olaparib.

TNBC cell lines MDA-MB-231, Hs578t, and MDA-MB-468 were treated with Olaparib only or in combination with one of the DDT pathway inhibitors (JHRE06, PF477736, B02) or with Verapamil. IC_50_-values (µM) were determined 48 h post-treatment. Olaparib was titrated, whereas the inhibitors were applied at constant IC_20_, whereby the mean was calculated from the respective monotreatments for all 3 cell lines and culture conditions (IC_20_ JHRE06 = 1.3 µM, IC_20_ PF477736 = 1.5 µM, IC_20_ B02 = 174.6 µM). Verapamil was used at a constant concentration of 50 µM. Cell viability was determined by MTT assay in 2D and 3D. IC_50_-values were calculated from combined viability data of twelve independent experiments performed in duplicates for Olaparib monotherapy and of three independent experiments performed in duplicates for Olaparib combination treatments. The heat-map shows differences in IC_50_-values, with dark grey indicating lower IC_50_ (higher sensitivity) and white indicating higher IC_50_ (lower sensitivity). Each value below the median was colored grey, each value above the median was colored white, and the median was set to white. Values marked with “>“ indicate that the IC_50_ exceeded the highest tested concentration and was therefore not calculated. Values marked with “<“ indicate that the viability (mean ± SEM) at the lowest Olaparib concentration falls below 50%, so an IC_50_ was not calculated. The extra sum of squares *F*-test comparing the fit of nonlinear least square regression models was used to test for differences between MTT viability curves. Statistical comparison (*p*-value) between viability curves for Olaparib mono- versus combination treatments of TNBC in 2D or 3D is listed below the IC_50_ value. n.a., statistical analysis not applicable, i.e., statistical analysis was not possible since the IC_50_ either exceeded the highest tested Olaparib concentration or the viability (mean ± SEM) at the lowest Olaparib concentration falls below 50%.

## Discussion

It is well established that EMT plays a key role in invasiveness and metastasis of BC [52] and that CSCs additionally drive tumor initiation [53, 54] and chemoresistance [20]. In this work, we show that TNBC lines, particularly when cultured in 3D, featured higher expression of EMT marker Vimentin, low expression of epithelial markers *KRT18* and *CDH1*, and hallmarks of CSCs, namely an elevated CD44/CD24 ratio and augmented Nestin levels [33, 34]. Previous studies showed that human stem and progenitor cells maintain slow fork speed to ensure genomic stability [12, 13, 55, 56]. Here, we show that the EMT/SC plasticity of BC cells enforces dynamic changes in the replication program, causing a slowdown of nascent DNA synthesis, which was neither due to oxidative stress nor stalling (Fig. 2). These observations were reminiscent of the findings by Ihle et al. [13] revealing p53-triggered fork deceleration in human SCs. However, all TNBC lines express oncogenic mutant p53 (Supplementary Table 1) a version of the protein unable to impinge on replication speed by fork remodeling [57, 58]. Nevertheless, DDT pathway activation in TNBC cells was revealed by augmented Ub-PCNA levels [9, 59], in combination with nuclear accumulation of ZRANB3 foci in 3D (Fig. 3). We propose that TNBC cells, despite compromised DDRs [5, 60] and mutant p53 [6], still protect their genome by fork slow-down through acquisition of CSC features (see model in Fig. 5b).

When dissecting DDT pathway usage, we revealed preferences depending on the culture conditions across TNBC lines (Table 1). In 2D, repriming was preferred by all three cell lines. Notably, CHK1i, which inhibits repriming [47], caused a replication speed slowdown in 2D to an extent similar to 3D. Smaller contributions to fast replication in 2D came from TLS, by different effectors in different cell lines (Fig. 5b). In 3D, slow DNA replication was ruled by fork reversal triggered by different effectors in different cell lines. Despite these preferences, our data suggests concomitant use of competing DDT pathways both in 2D and 3D. Thus, CHK1i reduced fork speed also in 3D, albeit less prominently than in 2D. Conversely, TLS not only contributed to replication elongation in 2D but also in 3D, e.g. in Hs578t after POLζi (Fig. 5b). Compensatory events may also affect track lengths. A previously described antagonistic relationship between PRIMPOL and TLS [15, 40, 43] might explain track lengths after *POLH* knockdown in 3D-cultured MDA-MB-231 or *POLI* knockdown in 2D-cultured MDA-MB-468 cells. Similarly, compensatory recruitment of alternative fork remodelers [59, 61] may rule over the opposite effects of the knockdown of *SMARCAL1* in 2D- versus 3D-cultured MDA-MB-468. *HLTF* knockdown in 2D versus 3D also reveals opposite effects ascribed to its translocase and E3-ligase for PCNA-polyubiquitination in 3D versus its PCNA-monoubiquitination-mediated promotion of TLS in 2D [44, 45]. Taken together, these findings show that despite the heterogeneity amongst TNBC cell lines and context-dependent dynamic changes, DDT pathway usages are common and targetable.

Sensitivities of TNBC lines to pharmacological inhibition of distinct DDT pathway targets indeed revealed a coherent picture of HR-independent cytotoxicities. First, RAD52i and RAD51i, inhibiting break-induced replication and HR, respectively, showed no or small effects on cell viability [9, 10]. Second, inhibitors of upstream DDR components ATR, MRE11, and DNA2 mostly showed moderate effects on viability. ATR enforces cell cycle arrest and suppresses origin firing [62]; together with DNA2/EXO1, it mediates end resection for HR after fork collapse [63] and Cisplatin resistance in 3D-cultured MCF-7 [64]. MRE11 generates ssDNA substrates required for ATR recruitment to DNA [63]. The observed effects argue against a central role of repair of broken forks in the protection from cytotoxicity and put the spotlight on DDT.

In all three cell lines in 2D and to a lesser extent also in 3D, we revealed lower IC_50_-values (≤20 µM) for CHK1i than for ATRi (Table 2). Notably, several studies provide mechanistic insight into roles of CHK1 in lesion bypass, distinct from checkpoint signaling downstream of ATR: (i) ATR inhibition as a single treatment was reported to not fully abrogate CHK1 activity, since CHK1 activation can occur through DNA-PK-mediated backup phosphorylation [65, 66]. (ii) CHK1 loss induces replication barriers compromising cell survival independently of excess origin firing [67, 68], an interpretation supported by our results with CDC7i and arguing against a significant impact of origin firing. (iii) CHK1 operates at stalled replication forks as a structural scaffold that stabilizes forks, binds PCNA, and promotes TLS independently of its kinase functions [68, 69]. (iv) CHK1 phosphorylates and recruits PRIMPOL to restart stalled forks [47]. Our results revealed a vital role of the CHK1 kinase activity in fork elongation of TNBC cells regardless of their stemness phenotype, which we therefore interpret as reliance on rapid DDT events, possibly including but not limited to PRIMPOL. Such a reliance may be instrumental for TNBC cells, which appear to be particularly dependent on fast lesion bypass at the fork, as they are compromised in DNA repair and fork remodeling [9, 12, 60] and in G1-checkpoint control [66].

Finally, POLζi most severely reduced viability (IC_50_-values ≤ 7 µM) in all three cell lines, with a consistently strong effect both in 2D and 3D. For comparison, targeting TLS-POLs POLη or POLɩ reached similar effectiveness only in Hs578t and MDA-MB-468 in 2D. Hypersensitivity towards POLζi is consistent with its virtuous roles in fork elongation, namely in gap filling after repriming [70], bypass of ribonucleotides and hard-to-replicate sites such as repetitive sequences [38] and its non-redundant role as extender POL in TLS [25]. Cancer cells under replication stress are particularly dependent on DDT mechanisms [7, 71], making targeting multiple DDT pathways by POLζi an attractive strategy. However, caution is advised as inactivation of POLζ increases chromosomal abnormalities, which may generate drug-resistance [38].

Olaparib as a single agent had no effect on the viability of the TNBC lines, which is consistent with their wild-type *BRCA1*/*2* status [7, 8]. Sensitization to PARPi by combined chemotherapeutic treatment was suggested, particularly for *TP53*-mutated cancers such as TNBC [72]. Here, combination treatment with low-dose POLζi or CHK1i sensitized all three lines to Olaparib, except 3D-cultured MDA-MB-231. PARP inhibition is known to prevent the restart of reversed forks [9], thereby potentially enforcing TLS and repriming, which in the combined treatment with POLζi [38, 70] or CHK1i [47, 68] are also compromised. Additionally, if replication forks collapse during the combined treatment, POLζi could prevent the reported POLζi-mediated D-loop extension [73] and CHK1i could prevent BRCA1 foci formation and RAD51 loading [74]. In fact, the combination of Olaparib and RAD51i achieved a similar sensitization pattern, though both drugs were fairly ineffective in mono-treatments. Such synthetic lethality can be explained by multiple roles of RAD51 in the maintenance of replication fork integrity and HR [9, 10]. Our results suggest that Olaparib resistance can be overcome by targeting DDT mechanisms. Notably, 3D-cultured MDA-MB-231 cells remained resistant to each one of the three combination treatments. These cells were special in showing the highest dependency on ZRANB3-mediated fork deceleration (Table 1), the highest level of the stemness marker CD44, highest nuclear ZRANB3 foci numbers, particularly in 3D, and the lowest level of the DNA damage marker ɣH2AX, again particularly in 3D with and without Olaparib treatment (Supplementary Fig. 7). Together with our previous study on ovarian CSCs [13] these observations strengthen the idea of a mechanistic link between acquisition of stemness features, ZRANB3-mediated fork reversal and chemoresistance.

This work identified DDT pathway components as exploitable vulnerabilities of TNBC cells. Limitations of this study are the sample size of 10 BC cell lines, with in-depth mechanistic analysis of only three TNBC cell lines. Moreover, the unavailability of inhibitory drugs targeting PRIMPOL or fork reversal enzymes made it necessary to inhibit components of the respective DDT pathways, i.e., CHK1 and PARP1, respectively. Finally, our study lacks patient data. Still, small molecule inhibitors targeting DDT pathway components have demonstrated increasing translational momentum; the CHK1i Prexasertib (LY2606368) and MK-8776 have already been evaluated in Phase I/II clinical trials across multiple solid tumors, including TNBC [7, 74] The USP1i KSQ-4279, which prevents de-ubiquitination of PCNA, has entered Phase I clinical testing in patients with advanced solid tumors harboring HR defects, with early data showing synergy with Olaparib [75]. Though POLζi has not yet entered the clinical stage, its toxicity and tolerability seem acceptable [26]. Whether DDT targeting as a monotherapy or in combination is an efficacious and tolerable choice to treat TNBC patients is a strategy that warrants further investigation.

## Materials and methods

### Cell culture

Characteristics of BC cell lines used in this study are summarized in Supplementary Table 1. The cell lines MDA-MB-453, T47D, MCF-7, MDA-MB-134-VI, MDA-MB-231, Hs578t, MDA-MB-468 and MDA-MB-436 were cultivated in DMEM (Gibco/Thermo Fisher Scientific, Waltham, MA, USA), supplemented with 10% FBS (PAN Biotech, Aidenbach, Germany), 1.4% L-Glutamine (Gibco/Thermo Fisher Scientific), 1% Penicillin–Streptomycin–Glutamine (Gibco/Thermo Fisher Scientific), 0.1% Insulin (Gibco/Thermo Fisher Scientific), 0.1% hEGF (Sigma-Aldrich/Merck, St. Louis, MO, USA) and 1% non-essential amino acids (Gibco/Thermo Fisher Scientific). BT474 cells were cultivated in RPMI 1640 (Gibco/Thermo Fisher Scientific) supplemented with 20% FBS, 1% L-Glutamine, 0.25% Insulin, and 1% Penicillin–Streptomycin–Glutamine. CTC-ITB-01 [76] cells were cultivated in DMEM supplemented with 10% FBS, 1% Penicillin–Streptomycin (Gibco/Thermo Fisher Scientific), 1% Insulin–Transferrin–Selenium-A (Gibco/Thermo Fisher Scientific), 0.2 µg/ml cholera toxin (Enzo Life Sciences, Farmingdale, NY, USA), 50 ng/ml hEGF, 10 ng/ml hBFGF (Sigma-Aldrich/Merck), and 0.1 µg/ml hydrocortisone (Sigma-Aldrich/Merck). Cells were incubated at 37 °C and 5% CO_2_ except for CTC-ITB-01, which was incubated at 37 °C and 5.5% O_2_, 5% CO_2_. For experiments in spheroid cultivation, cells were transferred to spheroid medium Ham’s F-12 (Gibco/Thermo Fisher Scientific) supplemented with 0.2% hEGF, 0.2% hBFGF, 0.25% Insulin and 2% B-27 supplement without Vitamin A (Gibco/Thermo Fisher Scientific) and ultra-low attachment plates 48 h before the start of the experiment and incubated at 37 °C and 5.5% O_2_, 5% CO_2_. BT474 and MCF-7 cells were provided by Cell Line Service, Eppelheim, Germany; MDA-MB-134-VI by American Type Culture Collection, Manassas, VI, USA; MDA-MB-468 by Leibniz Institute DSMZ-German Collection of Microorganisms and Cell Cultures GmbH, Braunschweig, Germany; MDA-MB-453, BT474, T47D, MDA-MB-134-VI, MDA-MB-231, Hs578t, and MDA-MB-436 by University Clinic Ulm, Germany. CTC-ITB-01 was newly established from circulating tumor cells of a metastatic breast cancer patient in our previous study [76]. All cell lines have been authenticated by STR profiling, and mycoplasma negativity was verified regularly.

### Drug treatment

For western blotting, FACS and RT-qPCR analysis, cells were treated with 10 µg/ml Carboplatin, 0.31 µM Doxorubicin, and 1 µM Olaparib for 8 days in total. The cells were treated on days 1, 3, and 6. In the DNA fiber assay, cells were treated with 5 mM NAC (160280250, Thermo Fisher Scientific) [77] for 4 h prior to the incorporation steps. For the DNA fiber assay, each cell line was treated for 4 h with the respective IC_50_ concentration for JH-RE-06, PF-477736, or Olaparib (10 µM) [78].

### Western blotting

Western blot analysis was performed as previously described in Hampp et al. 2016 [12]. For immunostaining the following antibodies were used: anti-Nestin (mouse mAB, MA1-110 Invitrogen/Thermo Fisher Scientific, Waltham, MA, USA, diluted 1:500 in 5% milk), anti-PCNA (mouse mAb, ab29, Abcam Limited, Cambridge, UK, diluted 1:1000 in 5% milk), anti-Pol Iota (rabbit pAb, A301-304A Bethyl, Montgomery, TX, USA, diluted 1:2000 in 5% milk), anti-Ubiquityl-PCNA (rabbit mAB, 13439 Cell Signaling Technology, Danvers, USA, diluted 1:1000 in 5% milk) and anti-Vimentin (rabbit pAb, 10366-1-AP, Proteintech Group, Inc., Chicago, IL, USA, diluted 1:1000 in 5% milk) and anti-phospho-Histone H2A.X (Ser139) (mouse mAb, 05-636, Sigma-Aldrich/Merck, diluted 1:1400 in 5% BSA). To control for protein loading, anti-GAPDH (mouse mAb, ab9484, Abcam Limited, diluted 1:2500 in 5% milk) and anti-alpha Tubulin (mouse mAb, ab7291, Abcam Limited, 1:5000 in 5% BSA) were used. Chemiluminescent detection and quantification were done in the linear range using ImageLab on a ChemiDocMP System (BioRad Laboratories, Munich, Germany).

### Reverse transcription and quantitative PCR (RT-qPCR)

Total RNA was isolated using the RNeasy^®^ Mini Kit (Qiagen, Hilden, Germany) according to the manufacturer’s instructions. The QuantiTect^®^ Reverse Transcription Kit (Qiagen) was used for reverse transcription. Quantitative PCR was performed as described in Eberle et al. 2021 [79] using the SensiFAST Probe LO-ROX kit (Bioline, London, UK). The PCR was performed on the qTower [3] 84G (Analytik Jena, Jena, Germany) using the program 2 min 95 °C and 40 cycles of 10 s 95 °C and 20 s 60 °C. Controls were ACTB HEX, PPIA HEX, and RPLA4 HEX (BioRad Laboratories). Genes of interest were analyzed with KRT18 FAM, CDH1 FAM, ZEB1 FAM, VIM FAM, POLH FAM, REV3L FAM, CCDC111 FAM, HLTF FAM, ZRANB3 FAM, SMARCAL1 FAM (BioRad Laboratories), and Pol Iota FAM (Thermo Fisher Scientific).

### Knockdown experiments

HiPerFect (Qiagen) was used for siRNA transfection for silencing targets in MDA-MB-231, Hs578t, and MDA-MB-468. FlexiTube GeneSolution siRNAs (Qiagen) with pools of four target-specific siRNAs were used to silence POLH (GS5429), POLI (GS11201), REV3L (GS5980), PRIMPOL/CCDC111 (GS201973), HLTF (GS6596), ZRANB3 (GS84083), and SMARCAL1 (GS50485), as established before [12,15]. Non-silencing (ns) siRNA (Qiagen) was used as a negative control. All siRNAs have been established in Castano et al. [15].

### DNA fiber spreading assay

Briefly, either 48 h post-spheroid seeding or 24 h post-2D seeding and 72 h post-transfection or 4 h post-inhibitor-treatment, the cells were pulse-labeled for 20 min with 20 µM 5-chloro-2’-deoxyuridine (CldU) (Sigma-Aldrich), washed with PBS, followed by a second 20 min pulse with 200 µM 5-iodo-2’-deoxyuridine (IdU) (Sigma-Aldrich). Cells were washed and harvested, either by trypsinization for adherent cells or centrifugation for 3D spheroid cells. Cells were resuspended in cold PBS and the concentration adjusted to 1250 cells/µl. 2 µl of cell suspension was subjected to lysis with 6 µl lysis buffer (0.5% SDS, 200 mM Tris–HCl, pH 7.4, 50 mM EDTA) directly on slides. Lysed cells were spread on the slides in a circular motion and then air-dried for 6 min. The slides were put at a 20° angle and dried again for 6 min. The slides were transferred into 3:1 methanol:acetic acid for 5 min, dried for 7 min and stored in 70% (v/v) ethanol at 4 °C overnight. Slides were incubated in 2.5 N HCl for denaturation/deproteination for 1 h at RT. Slides were blocked with 5% BSA for 45 min, followed by immunofluorescent staining. Slides were incubated with primary antibodies anti-BrdU (rat mAb, 426326, Abcam, 1:140) for IdU detection and anti-BrdU (mouse mAb, 347580 BD Biosciences, Franklin Lakes, NJ, USA, 1:70) for CldU detection for 1 h at RT, followed by secondary antibodies AlexaFluor™ 555 anti-mouse (Invitrogen/Thermo Fisher Scientific, 1:250) and AlexaFluor™ 488 anti-rat (Invitrogen/Thermo Fisher Scientific, 1:400) for 1 h at RT. The slides were imaged using the Keyence BZ-9000/BZ-X800 microscope with the ×40 objective. Fibers were measured with the Fiji (which is just ImageJ) software.

### Viability assay

Cell survival was determined by MTT assay (3-(4,5-dimethythiazol-2-yl)-2,5-diphenyl tetrazolium bromide) (Sigma-Aldrich/Merck), described previously [23]. Prior to drug treatment, cells were seeded in 96-well plates at a density of 4 × 10^4^ cells per well. Treatments were applied 48 h after seeding in 3D spheroid culture media and 24 h after seeding in 2D monolayer culture. The MTT assay was performed after 48 h of treatment. First, the MTT was dissolved in 1xPBS (Gibco/Thermo Fisher Scientific) to an end concentration of 5 mg/ml. This MTT solution was diluted in DMEM 1x without phenol red (Gibco/Thermo Fisher Scientific) to a final concentration of 1 mg/ml. 100 µl of this solution was added to each well and then incubated for 2.5 h at 37 °C. MTT was removed after the incubation time, and the cells were resuspended in 200 µl 5% HCl/95% isopropanol (Sigma-Aldrich/Merck). The plates were shaken for 10 min and the optical density was measured at 570 nm with a Tecan Sunrise Photometer (Tecan, Crailsheim, Germany). The MTT experiments were conducted in three independent experiments with duplicates each. IC_50_-values were calculated using GraphPad Prism 9 software (GraphPad Software Inc., La Jolla, CA, USA). For details of drugs and concentrations, see Supplementary Materials and Methods.

### Flow cytometric analysis of stemness markers

Cells were harvested, washed, and fixed with 4% formaldehyde for 10 min, washed again, transferred to a 96-well plate, and stained with FITC anti-CD24 (mouse mAb, 555427 BD Biosciences, diluted 1:10 in 2% FBS/PBS) or FITC anti-CD44 (mouse mAb, 555478, BD Biosciences, diluted 1:10 in 2% FBS/PBS) overnight at 4 °C. Cells were washed and resuspended in 200 µl 2% FBS/PBS and analyzed in a FACS Calibur flow cytometer (BD Biosciences). Mean fluorescence intensities were measured in the FL1/FL2 plot following live cell SSC/FSC gating. MCF12a (2D, untreated) was used as an internal standard to subtract background signals for CD44 and CD24, respectively. Samples were measured in triplicate, in two independent experiments.

### Immunofluorescence microscopy

For ɣH2AX foci analysis, MDA-MB-231 cells were treated for 48 h with 10 µM Olaparib 48 h post 3D spheroid or 24 h post 2D seeding. 2D-cultured cells were seeded on four-well chamber slides (Corning Inc., Corning, NY, USA), and 3D spheroid cells were harvested and spun onto Poly-L-lysine-covered slides. Pre-extraction was performed for 1 min in 20 nM HEPES, 50 mM NaCl, 1 mM EDTA, 3 mM MgCl_2_, 300 mM Sucrose, 0.5% (v/v) Triton X-100, followed by fixation with 3.6% formaldehyde for 10 min. Cells were washed three times with 0.5% Triton-PBS, blocked with 5% BSA for 1 h at room temperature, and then incubated with primary antibodies against anti-phospho-Histone H2A.X (Ser139) (mouse mAb, 05-636, Sigma-Aldrich/Merck, diluted 1:1000 in 5% BSA) overnight at 4 °C. Cells were washed three times with 0.5% Triton-PBS and incubated with secondary anti-mouse (Alexa Fluor™ 488 goat anti-mouse, A-11001, Invitrogen/Thermo Fisher Scientific, diluted 1:2000 in PBS) for 45 min at 37 °C. Cells were washed and mounted with VectaShield^®^ containing DAPI. The slides were subjected to high-content imaging with an Axio Observer 7 microscope (Carl Zeiss AG, Oberkochen, Germany), objective Plan-Apochromat ×100/1.4 Oil DIC M27, and automated analysis with Zeiss ZEN 3.1 (blue edition) software (Carl Zeiss AG).

For colocalization analysis, 2D-cultured cells were seeded on four-well chamber slides (Corning Inc.), and 3D spheroid cells were harvested and spun onto Poly-L-lysine-covered slides, pre-extracted, fixed, washed, and blocked as described above. Then, the slides were incubated with primary antibodies against POLι (POLI Monoclonal Antibody, MA5-26503, Invitrogen/Thermo Fisher Scientific, diluted 1:150 in 5% goat serum) and ZRANB3 (ZRANB3 Polyclonal antibody, 23111-1-AP, Proteintech Group, Inc., diluted 1:50 in 5% goat serum) for 1 h at 37 °C. Cells were washed three times with PBS and incubated with secondary anti-mouse (Alexa Fluor™ 488 donkey anti-rabbit, A-21202, Invitrogen/Thermo Fisher Scientific, diluted 1:2000 in PBS) and anti-rabbit (Alexa Fluor™ 594 goat anti-rabbit, A-11037, Invitrogen/Thermo Fisher Scientific, diluted 1:1000 in PBS) for 45 min at 37 °C. Cells were mounted with VectaShield^®^ containing DAPI and subjected to high-content imaging as detailed above.

### Statistical analysis

A graphic presentation of data was performed using GraphPad Prism 9/10 software (La Jolla, CA, USA). For *k*-sample cases, we first performed Kruskal–Wallis *H*-tests, which, in case of statistical significance, were followed by two-tailed Mann–Whitney *U* tests for post-hoc pairwise comparisons. MTT viability curves were plotted and IC_50_ values were calculated using GraphPad Prism 9/10 software. The extra sum-of-squares *F*-test comparing the fit of nonlinear least-square regression models was used to test for differences between MTT viability curves. A *p*-value < 0.05 was considered statistically significant, and there was no adjustment of the significance level for multiple comparisons.

## Supplementary information

Supplementary Figures

Supplementary Tables

Supplementary text

Source data

## Data Availability

The datasets generated during and/or analyzed during the current study are available from the corresponding author on reasonable request.
