# Supplementary material for: Targeting common denominators of DNA damage tolerance mechanisms as therapeutic strategy to cope with dynamic phenotypic changes in triple-negative breast cancer cells
Source: Cell Death Discov. 2026 Oct 6;12:405. doi: 10.1038/s41420-026-03377-4 (PMC13642338; doi:10.1038/s41420-026-03377-4)
Supplement: Supplementary file 1 [file 41420_2026_3377_MOESM1_ESM.pdf]

a

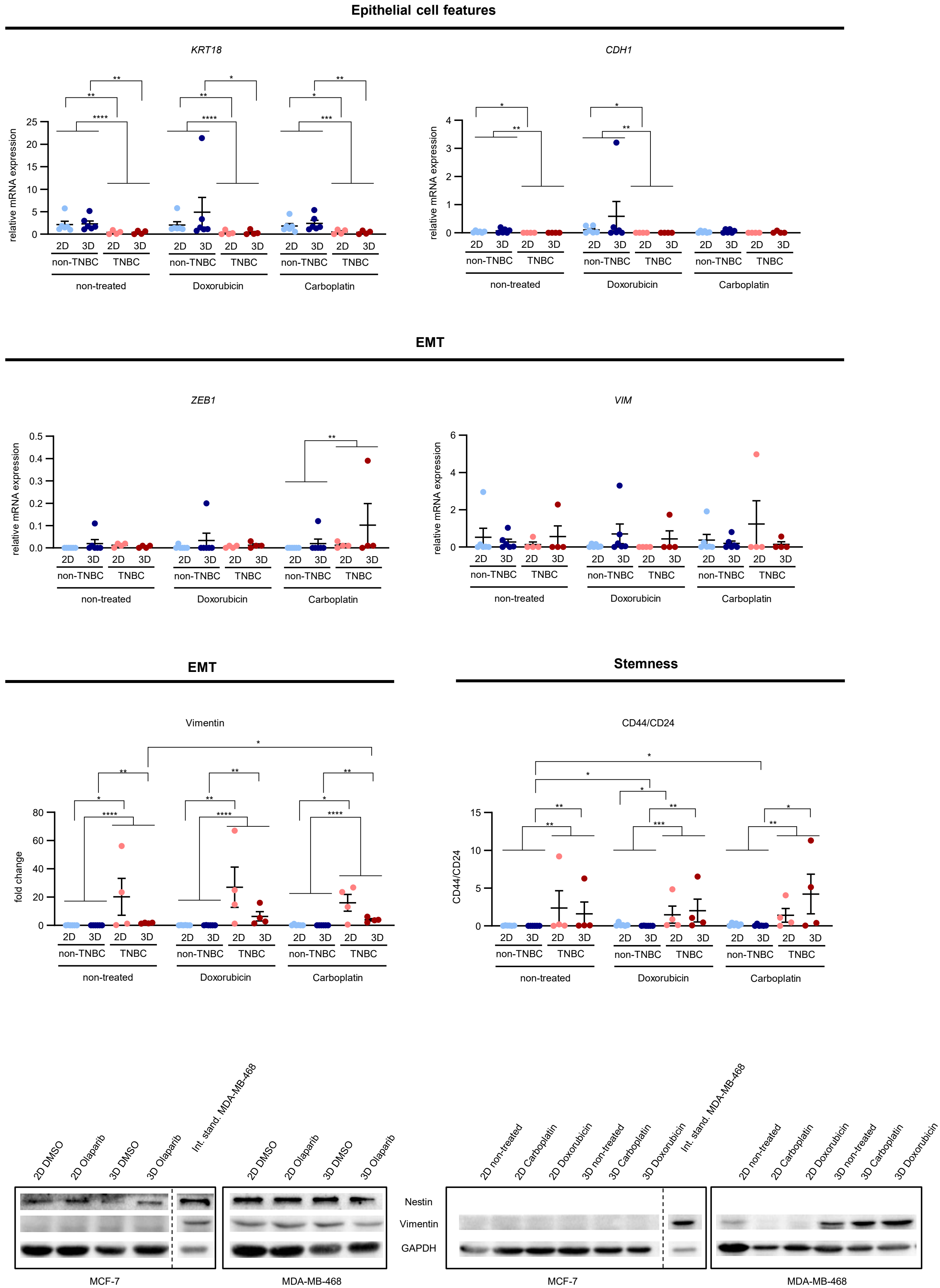
Epithelial cell features
KRT18
CDH1
relative mRNA expression
2D
3D
non-TNBC
TNBC
non-treated
Doxorubicin
Carboplatin
EMT
ZEB1
VIM
EMT
Vimentin
fold change
Stemness
CD44/CD24
b
2D DMSO
2D Olaparib
3D DMSO
3D Olaparib
Int. stand. MDA-MB-468
Nestin
Vimentin
GAPDH
2D non-treated
2D Carboplatin
2D Doxorubicin
3D non-treated
3D Carboplatin
3D Doxorubicin
MCF-7
MDA-MB-468

c

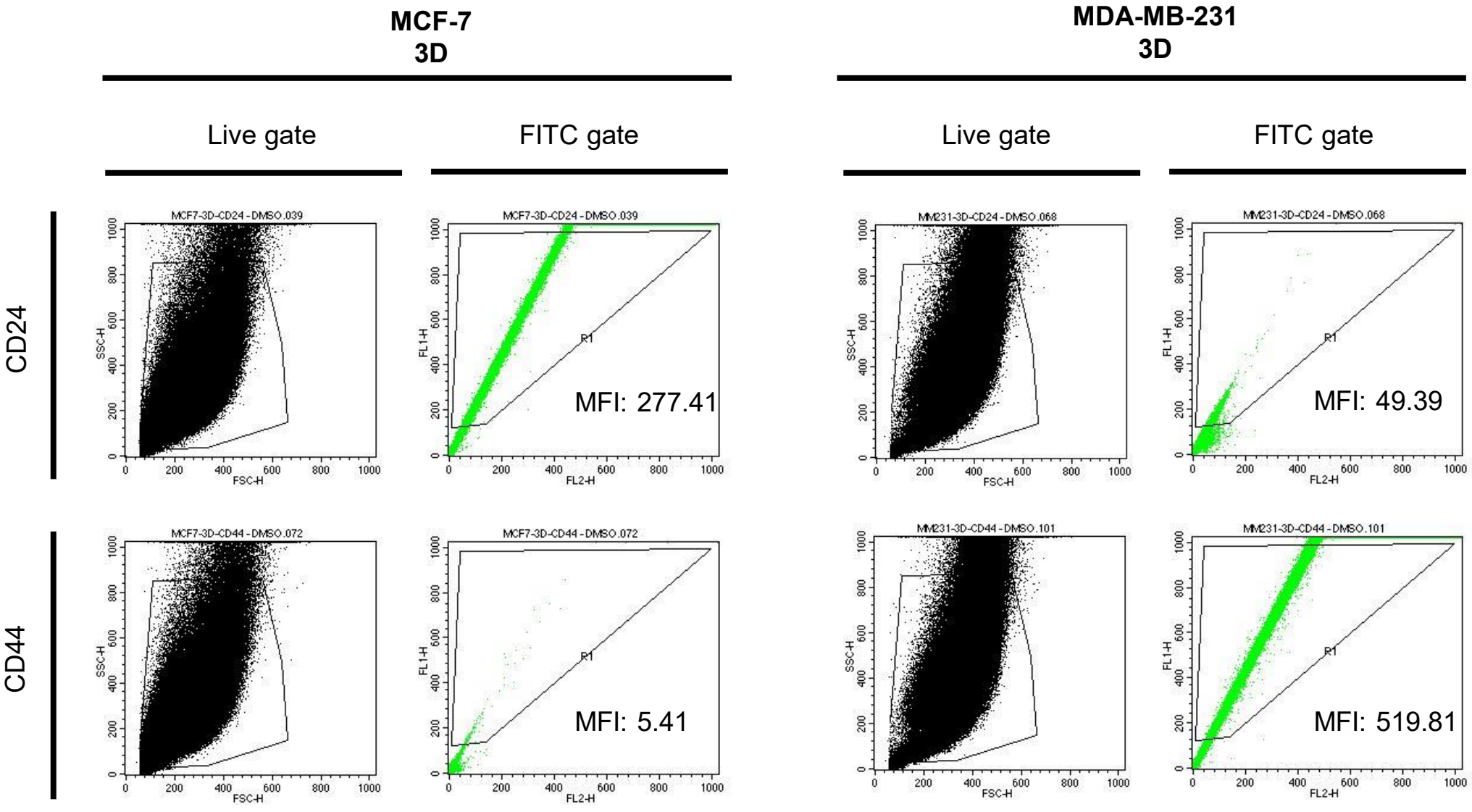

Supplementary Figure 1. Analysis of epithelial cell, EMT and stemness features of non-TNBC and TNBC cell lines with and without Doxorubicin and Carboplatin treatment.

a Cells were treated for eight days before RT-qPCR, western blot and FACS analysis, with the drugs (Doxorubicin: 0.31 µM, Carboplatin: 10 µg/ml) or H_2O (non-treated) being administered every two to three days (see Figure 1a). Upper panels with epithelial cell features: Relative mRNA expression levels for *KRT18* and *CDH1* are shown. Middle panels with EMT features: Relative mRNA expression levels of *ZEB1* and *VIM*. mRNA expression levels were determined by RT-qPCR, normalized to at least two of three housekeeping genes (*ACTB*, *RPL4*, *PPIA*) with the $2^{-\Delta Ct}$ method. Lower panels with EMT or stemness features: Relative protein levels. Vimentin levels were determined by western blot analysis and normalized to GAPDH and internal standard MDA-MB-468 sample (2D, untreated) from the same blot. Ratio of CD44/CD24 was calculated from mean fluorescent intensities of CD44 and CD24 measured by FACS in the live cell gate in respective samples. Dots show the mean values from two to three independent experiments (N=2-3), with each dot representing a single cell line. Blue dots for non-TNBC, red for TNBC with light blue and light red for 2D, and dark blue and dark red for 3D cultivation. Non-TNBC: MDA-MB-453, BT474, T47D, MCF-7, MDA-MB-134-VI, CTC-ITB-01. TNBC: MDA-MB-231, Hs578t, MDA-MB-468 and MDA-MB-436. Bars and whiskers indicate mean values ± SEM of the means for different cell lines. Significances were calculated by Mann-Whitney U test, in case of statistical significance being reached with Kruskal-Wallis H-test, using GraphPad Prism 9. *: P<0.05; **: P<0.01; ***: P<0.001; ****:P<0.0001.

b Western blots are representatively shown for the non-TNBC line MCF-7 and the TNBC line MDA-MB-468. Note that the GAPDH blots shown for the respective cell lines are the same loading controls as used in Supplementary Figure 2c. For uncropped Western blots see Source Data.

c Exemplary FACS plots of MCF-7 and MDA-MB-231in 3D under DMSO treatment, showing the live cell SSC/FSC and the subsequent FL1/FL2 gating for CD24 and CD44 mean fluorescent intensities. MFI= mean fluorescent intensities.

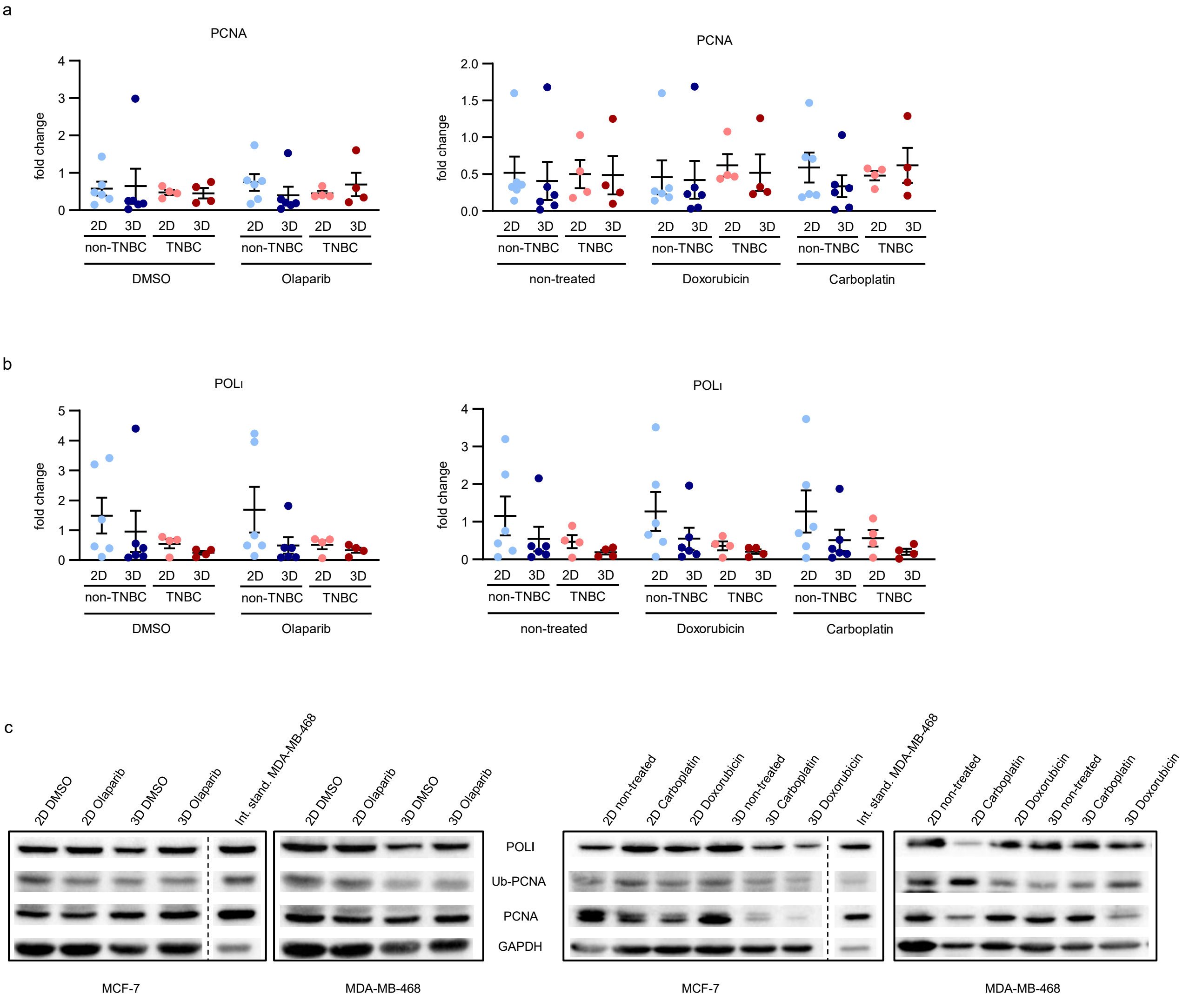

Supplementary Figure 2. Western blot analysis of PCNA and POLι

a, **b** Comparison of the relative protein levels of PCNA and POLι in non-TNBC and TNBC cell lines under DMSO and Olaparib (1 µM) treatment, H_2O (non-treated), Doxorubicin (0.31 µM) and Carboplatin (10 µg) treatment. The protein levels were determined by western blot analysis and normalized to GAPDH and internal standard (Int. stand.) MDA-MB-468 (2D, non-treated). Dots show the mean value from two independent experiments (N=2), with each dot representing a single cell line. Blue dots for non-TNBC cell lines, red for TNBC cell lines with light blue and light red for 2D, and dark blue and dark red for 3D cultivation. Non-TNBC: MDA-MB-453, BT474, T47D, MCF-7, MDA-MB-134-VI, CTC-ITB-01. TNBC: MDA-MB-231, Hs578t, MDA-MB-468 and MDA-MB-436. Bars and whiskers indicate mean values ± SEM of the means for different cell lines. Significances were calculated by Mann-Whitney U test, in case of statistical significance being reached with Kruskal-Wallis H-test, using GraphPad Prism 9. **c** Western blots are shown for MCF-7 and MDA-MB-468. Note that the GAPDH blots shown for the respective cell lines are the same loading controls as used in Supplementary Figure 1b. For uncropped Western blots see Source Data.

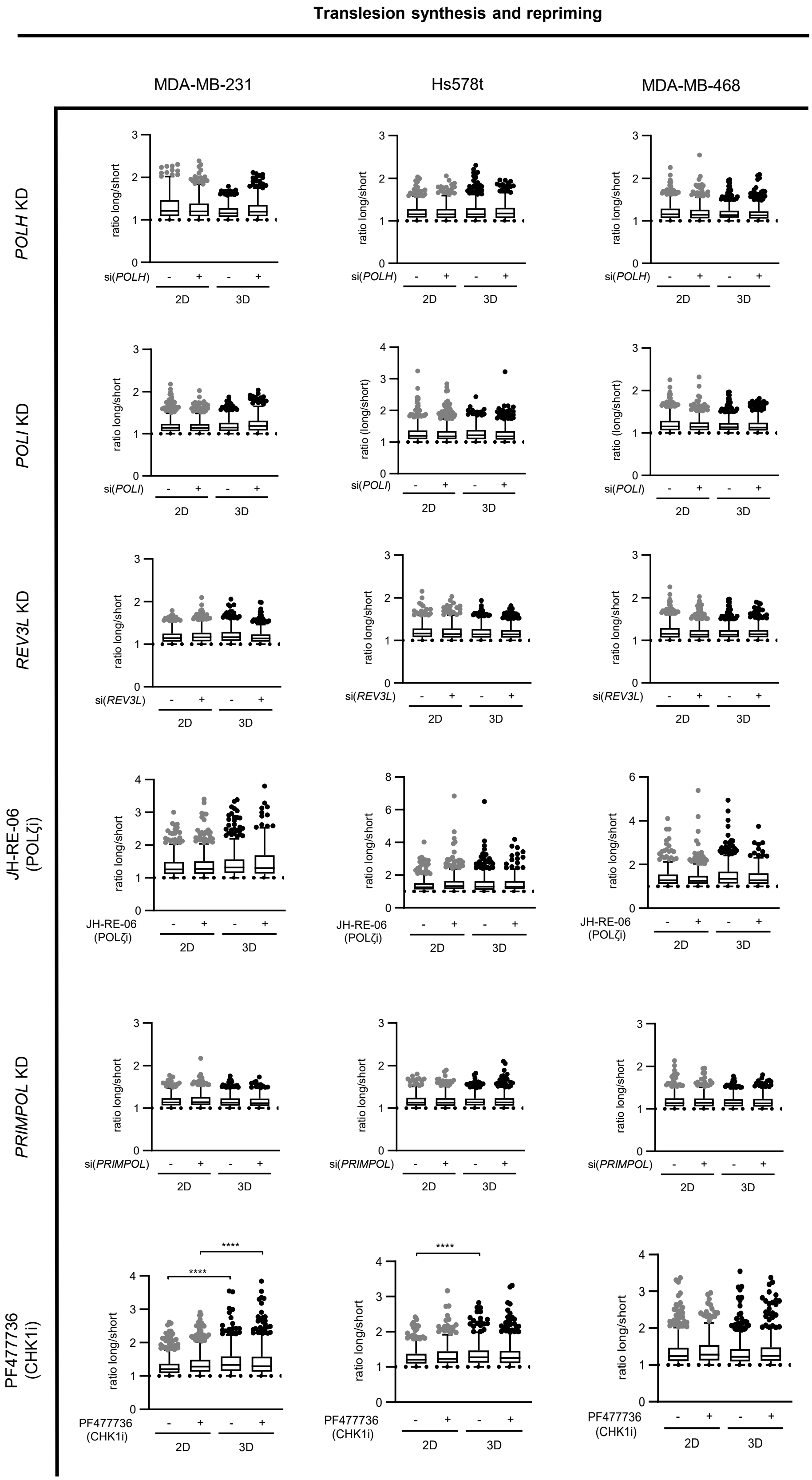

Supplementary Figure 3: Analysis of fork stalling after targeting translesion synthesis (POLH, POLI, REV3L, POLζ) and repriming (PRIMPOL, CHK1).

Comparison of the ratios of long/short tracks (Mean ± SEM from ≥375 fibers from three to four individual experiments) in 2D (grey) and 3D (black) after knockdown of *POLH*, *POLI*, *REV3L* and *PRIMPOL* (10 nM siRNA each) or inhibition of POLζ with JH-RE-06 and CHK1 with PF477736 using the respective IC50 concentration (Table 2) in MDA-MB-231, Hs578t and MDA-MB-468. Significances were calculated by Mann-Whitney U test, in case of statistical significance being reached with Kruskal-Wallis H-test, using GraphPad Prism 9. Data is shown in box plots (50%) including median (line), 95% confidence intervals. ****: P<0.0001.

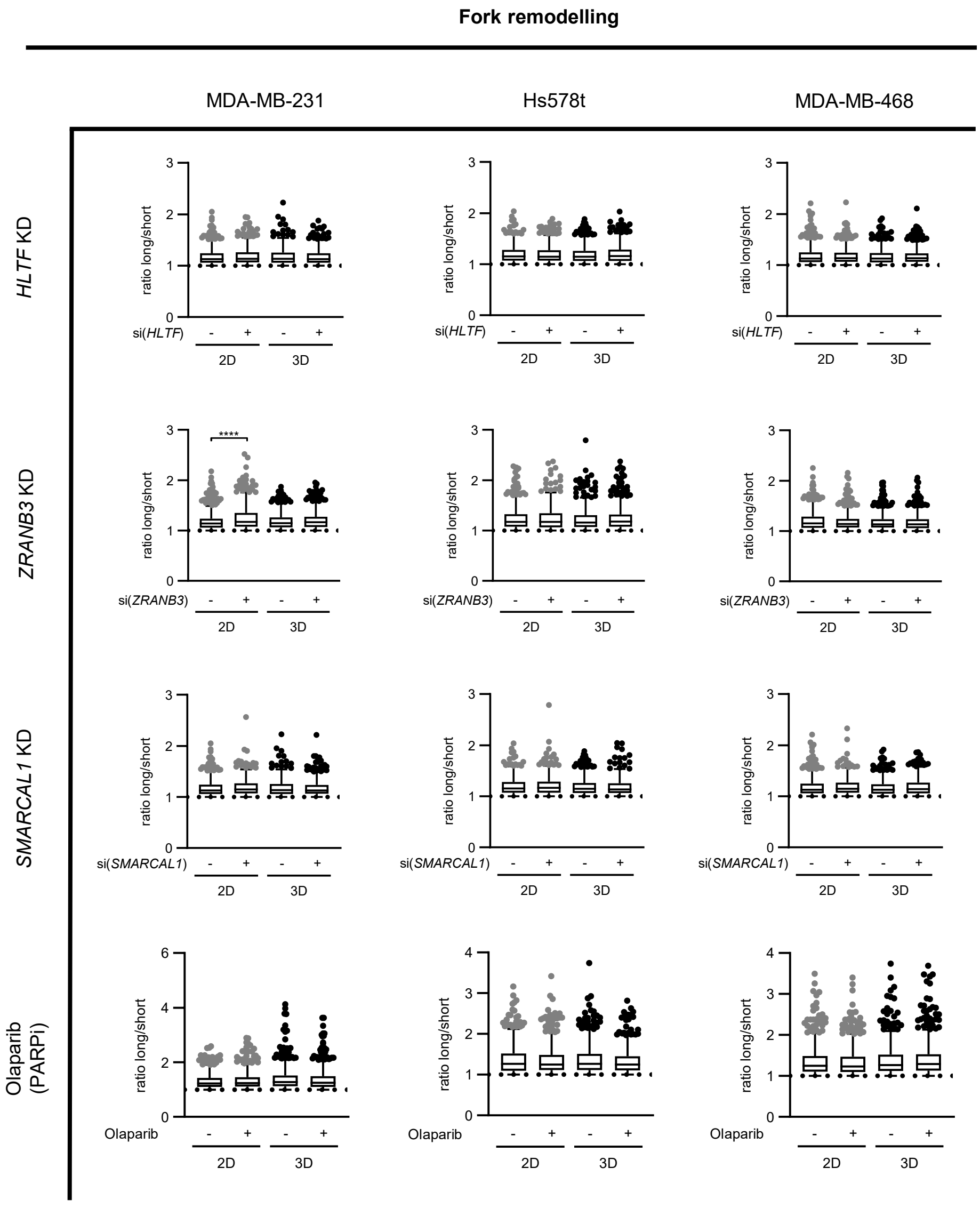

Supplementary Figure 4: Analysis of fork stalling after targeting fork remodeling (HLTF, ZRANB3, SMARCAL1, PARP).
Comparison of the ratios of long/short tracks (Mean ± SEM from ≥516 fibers from three to four individual experiments) in 2D (grey) and 3D (black) after knockdown of *HLTF*, *ZRANB3* and *SMARCAL1* (10 nM siRNA each) and inhibition of PARP with Olaparib (10µM) in MDA-MB-231, Hs578t and MDA-MB-468. Significances were calculated by Mann-Whitney U test, in case of statistical significance being reached with Kruskal-Wallis H-test, using GraphPad Prism 9. Data is shown in box plots (50%) including median (line), 95% confidence intervals. ****: $P<0.0001$.

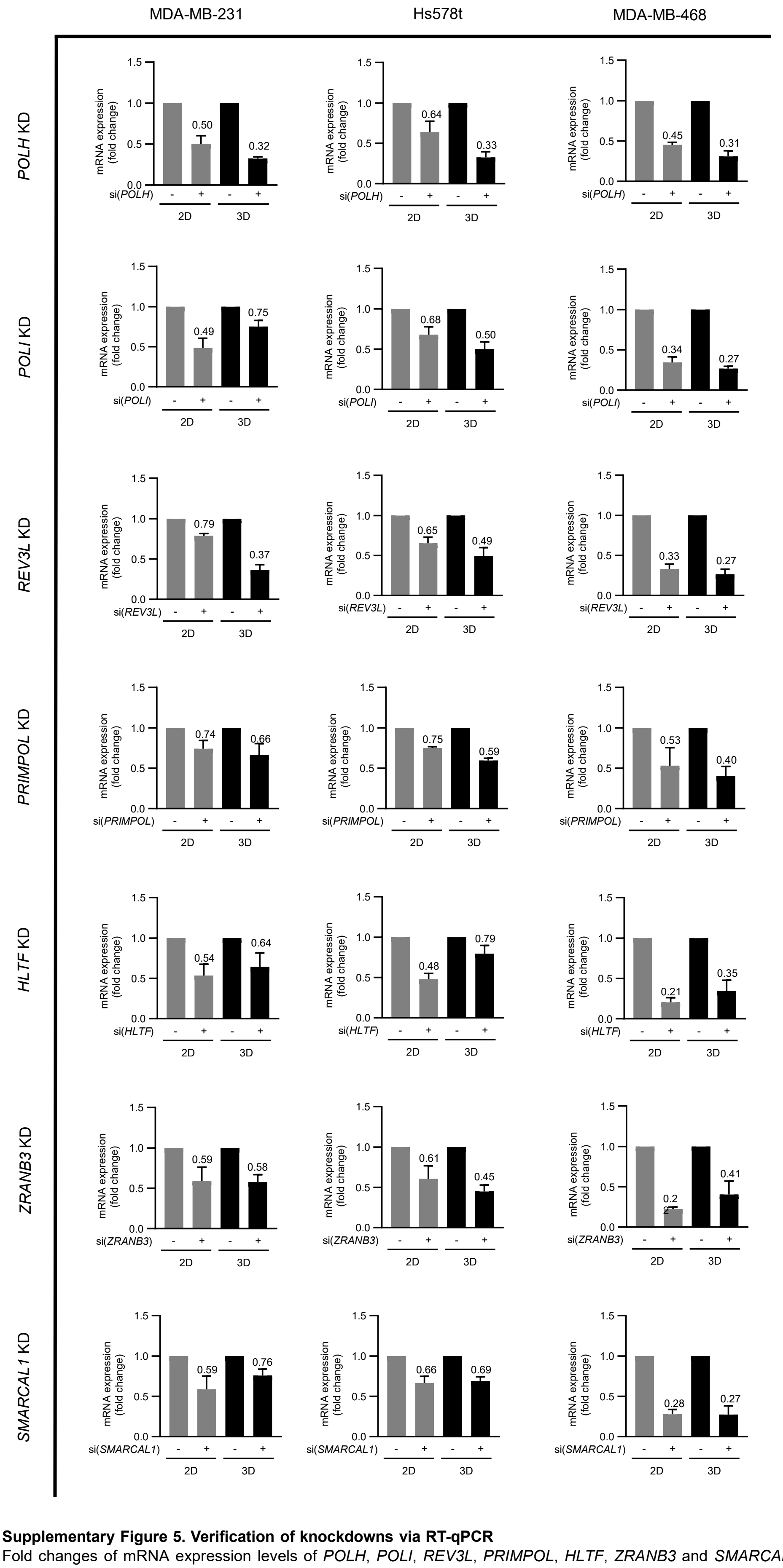

Supplementary Figure 5. Verification of knockdowns via RT-qPCR

Fold changes of mRNA expression levels of *POLH*, *POLI*, *REV3L*, *PRIMPOL*, *HLTF*, *ZRANB3* and *SMARCAL1* in 2D (grey) and 3D (black). mRNA expression levels were determined with RT-qPCR, normalized to at least two out of three housekeeping genes (ACTB, RPL4, PPIA) and to the respective controls via the $2^{-\Delta\Delta Ct}$ method measurements in duplicates in three individual experiments (N= 3). Mean ± SEM.

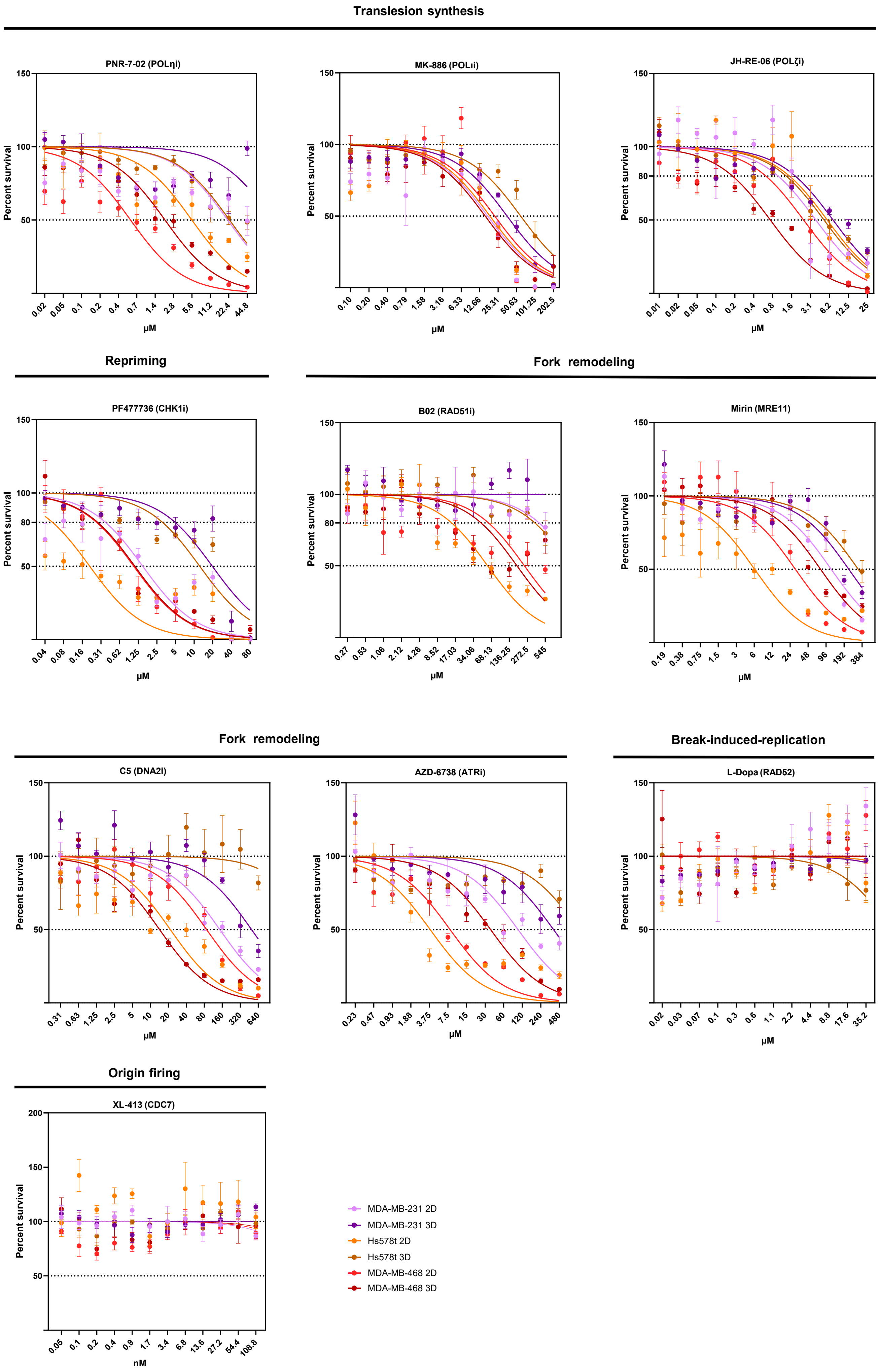
Translesion synthesis
PNR-7-02 (POLηi)
MK-886 (POLιi)
JH-RE-06 (POLζi)
Repriming
PF477736 (CHK1i)
Fork remodeling
B02 (RAD51i)
Mirin (MRE11)
Fork remodeling
C5 (DNA2i)
AZD-6738 (ATRi)
Break-induced-replication
L-Dopa (RAD52)
Origin firing
XL-413 (CDC7)
Percent survival
μM
nM
MDA-MB-231 2D
MDA-MB-231 3D
Hs578t 2D
Hs578t 3D
MDA-MB-468 2D
MDA-MB-468 3D

Supplementary Figure 6. Viability curves of TNBC cell lines for treatment with different DDT pathway inhibitors

TNBC cell lines MDA-MB-231, Hs578t and MDA-MB-468, cultivated in 2D and 3D, were treated for 48 h with different concentrations of PNR-7-02, MK-886, JH-RE-06, PF477736, B02, Mirin, C5, AZD-6738, L-Dopa and XL-413. MTT viability curves were generated by GraphPad Prim version 9. Each data point represents the mean from three independent experiments with duplicates each. Mean ± SEM.

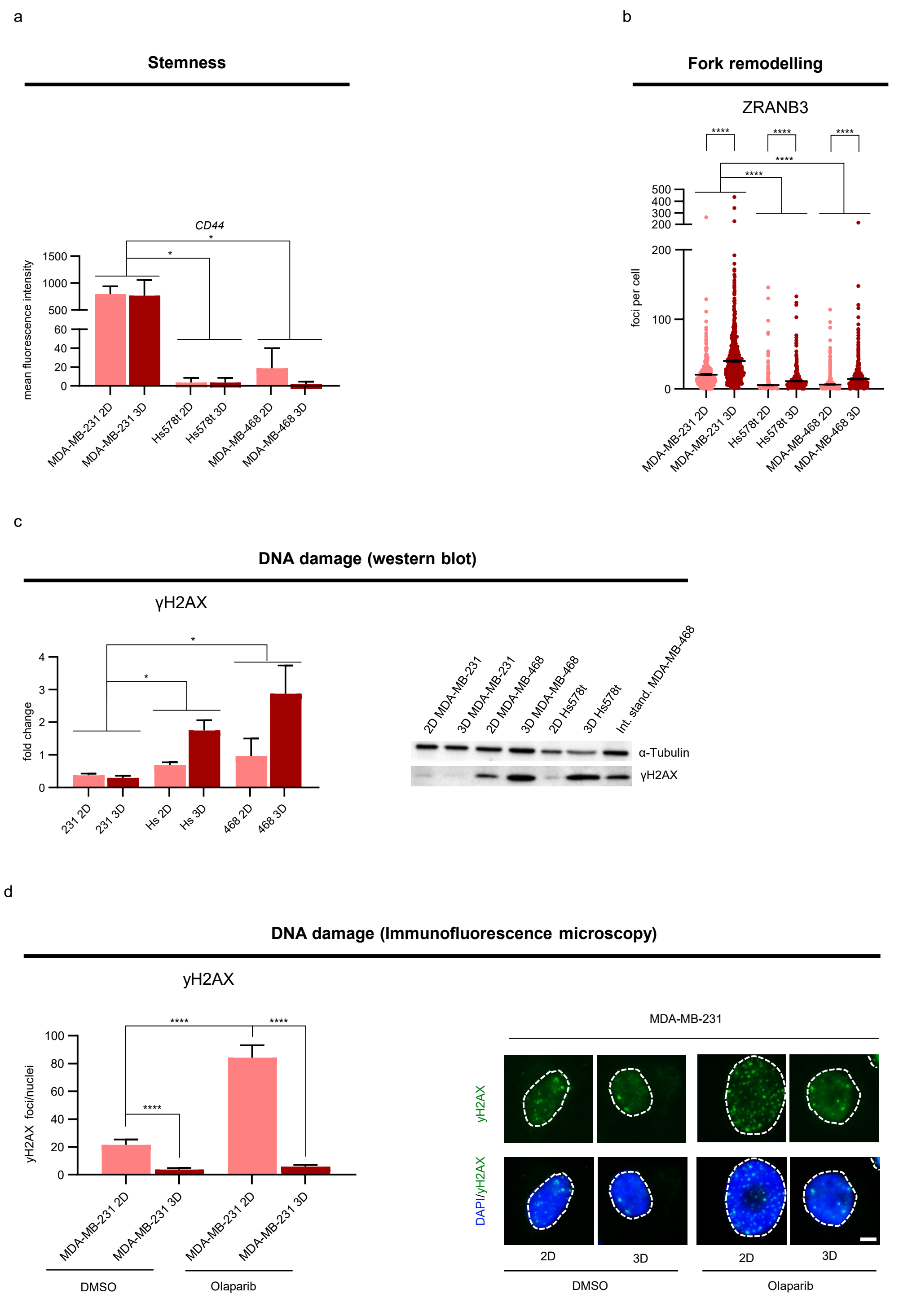

Supplementary Figure 7. Analysis of stemness marker CD44, fork remodelling component ZRANB3 and γH2AX-labeled DNA damage in MDA-MB-231 versus Hs578t and MDA-MB-468 cells.

a Comparison of mean fluorescent intensity of CD44 in MDA-MB-231, Hs578t and MDA-MB-468 cells. Bars indicate mean values ± SD, N=2, n=4

b Comparison of ZRANB3 foci per cell in MDA-MB-231, Hs578t and MDA-MB-468. Bars indicate mean values ± SEM, N=2, n=1563-1631

c Comparison of the relative levels of γH2AX in non-treated MDA-MB-231, Hs578t and MDA-MB-468 cells as determined by western blotting. Bars indicate mean values ± SD. N=2. Light red for 2D, dark red for 3D. Representative western blot is presented on the right. For uncropped Western blots see Source Data.

d γH2AX foci formation in 2D- and 3D-cultured MDA-MB-231 cells. After treatment with DMSO or Olaparib for 48 h cells were subjected to immunofluorescence microscopy. The left panel graphically displays nuclear γH2AX foci numbers, whereby bars indicate mean values ± SEM, N=2, n=23-27. Light red for 2D, dark red for 3D. On the right side representative images depicting green γH2AX foci and γH2AX foci in DAPI-stained nuclei are shown. Scale bar equals 5 μM.

All Significances were calculated by Mann-Whitney U test, in case of statistical significance being reached with Kruskal-Wallis H-test, using GraphPad Prism 9, *: P<0.05, ****: P<0.0001.
