# Supplementary material for: Targeting common denominators of DNA damage tolerance mechanisms as therapeutic strategy to cope with dynamic phenotypic changes in triple-negative breast cancer cells
Source: Cell Death Discov. 2026 Oct 6;12:405. doi: 10.1038/s41420-026-03377-4 (PMC13642338; doi:10.1038/s41420-026-03377-4)
Supplement: Supplementary file 2 [file 41420_2026_3377_MOESM2_ESM.pdf]

Supplementary Table 1. Characteristics of breast cancer cell lines used in this study.

Cell line/features	MDA-MB-453	BT474	T47D	MCF-7	MDA-MB-134-VI	CTC-ITB-01	MDA-MB-231	Hs578t	MDA-MB-468	MDA-MB-436
**ER status*[1]**	-	+	+	+	+	+	-	-	-	-
**PR status[1]**	-	+	+	+	-	+	-	-	-	-
**HER2 status[1]**	+	+	-	-	-	low	-	-	-	-
***TP53* status**	Ex10-11del[2]	p.Glu285Lys[3]	p.Leu194Phe[4]	WT[5]	p.Glu285Lys[6]	p.Glu285Lys[7]	p.Arg280Lys[8]	p.Val157Phe[9]	p.Arg273His[10]	p.Glu204fs*7[11]
***BRCA1* status[1]**	WT	WT	WT	WT	WT	WT	WT	WT	WT	c.5277+1G>A
**Subtype[1]**	H	LB	LA	LA	LA	LA/B	TN	TN	TN	TN
**Tumor[1]**	AC	IDC	IDC	IDC	IDC	ILC/IDC	AC	IDC	AC	AC

Legend to Supplementary Table 1. Characteristics of breast cancer cell lines used in this study.
ER, estrogen receptor; PR, progesteron receptor; HER2, HER2-positive; WT, wild-type; H, HER2-positive breast cancer; LA, luminal A; TN, triple-negative; AC, adenocarcinoma; IDC, invasive ductal carcinoma; ILC, invasive lobular carcinoma.

Supplementary Table 2. Statistics of differences between IC50-values determined in TNBC cell lines cultivated in 2D versus 3D using different DDT pathway inhibitors.

	p-value									
**DDT pathway**	**Translesion synthesis**			**Repriming**	**Fork remodelling**				**Break-induced replication**	**Origin firing**
**Inhibitor**	**PNR-7-02**	**MK-886**	**JH-RE-06**	**PF477736**	**B02**	**Mirin**	**C5**	**AZD-6738**	**L-Dopa**	**XL-413**
**Target**	POLη	POLι	POLζ	CHK1	RAD51	MRE11	DNA2	ATR	RAD52	CDC7
**Cell line/culture**	**2D** / **3D**	**2D** / **3D**	**2D** / **3D**	**2D** / **3D**	**2D** / **3D**	**2D** / **3D**	**2D** / **3D**	**2D** / **3D**	**2D** / **3D**	**2D** / **3D**
**MDA-MB-231**	n.a.	0.001	0.0204	<0.0001	n.a.	0.0014	<0.0001	<0.0001	n.a.	n.a.
**Hs578t**	<0.0001	<0.0001	0.6285	<0.0001	n.a.	<0.0001	n.a.	n.a.	n.a.	n.a.
**MDA-MB-468**	<0.0001	0.1125	<0.0001	0.8238	0.4755	<0.0001	<0.0001	<0.0001	n.a.	n.a.

Legend to Supplementary Table 2. Statistics of differences between IC50-values determined in TNBC cell lines cultivated in 2D versus 3D using different DDT pathway inhibitors.
IC50-values were calculated from the combined viability data obtained in three independent experiments with duplicates each (see Table 2). The extra sum of squares F-test comparing the fit of nonlinear least square regression models was used to test for differences between MTT viability curves (2D versus 3D). Shades of red indicate statistically significant differences (light red: $p<0.05$; dark red: $p<0.0001$). n.a., not applicable, i.e. statistical analysis was not possible since the IC50 exceeded the highest tested drug concentration.

Supplementary Table 3. Sensitivity of TNBC cell lines to Olaparib, Doxorubicin and Carboplatin and statistical comparisons between IC50-values in 2D versus 3D.

IC50 [µM]						
**drug**	**Olaparib**		**Doxorubicin**		**Carboplatin**	
**Cell line/culture**	**2D**	**3D**	**2D**	**3D**	**2D**	**3D**
**MDA-MB-231**	580.4	> 1000.0	6.4	15.8	> 431.0	> 431.0
P-value	n.a		0.0059		n.a	
**Hs578t**	183.6	> 1000.0	9.2	50.3	> 431.0	> 431.0
P-value	n.a		<0.0001		n.a	
**MDA-MB-468**	293.6	> 1000.0	0.3	0.9	14.7	10.6
P-value	n.a		<0.0001		0.1137	

Supplementary Table 3. Sensitivity of TNBC cell lines to Olaparib, Doxorubicin and Carboplatin and statistical comparisons between IC50-values in 2D versus 3D.
TNBC cell lines MDA-MB-231, Hs578t and MDA-MB-468 were treated with different concentrations of Olaparib, Doxorubicin and Carboplatin. Cell viability was determined 48 h post treatment in 2D- and 3D-cultivation by MTT assay. IC50-values (µM) were determined from combined viability data of three independent experiments performed in duplicates each. The extra sum of squares F-test comparing the fit of nonlinear least square regression models was used to test for differences between MTT viability curves (2D versus 3D). n.a., not applicable, i.e. statistical analysis was not possible since the IC50 exceeded the highest tested concentration.
