# Supplementary material for: Targeting common denominators of DNA damage tolerance mechanisms as therapeutic strategy to cope with dynamic phenotypic changes in triple-negative breast cancer cells
Source: Cell Death Discov. 2026 Oct 6;12:405. doi: 10.1038/s41420-026-03377-4 (PMC13642338; doi:10.1038/s41420-026-03377-4)
Supplement: Supplementary file 3 [file 41420_2026_3377_MOESM3_ESM.pdf]

Supplementary Material

Targeting common denominators of DNA damage tolerance mechanisms as therapeutic strategy to cope with dynamic phenotypic changes in triple-negative breast cancer cells

Kathrin Niedermayer, Sara Greco, Anke Oechsle, Tanja Köhler, Klaus Pantel, Thomas W.P. Friedl, Wolfgang Janni, Brigitte Rack, Kerstin Pfister, Vanesa Gottifredi, and Lisa Wiesmüller.

Table of contents:

- Supplementary Materials and Methods
- Supplementary References

Supplementary Materials and Methods

Viability assay

The cells were treated with the respective drugs in previously published concentration ranges as follows: 44.8 – 0.04 µM PNR-7-02[12] (Axon Medchem LLC, VA, USA); 202.5 - 0.1 µM MK-886[13] (Biomol GmbH, Hamburg, Germany); 25 - 0.02 µM JH-RE-06[14] (Axon Medchem LLC, VA, USA); 545 - 0.5 µM B02[15] (MedChemExpress LLC, NJ, USA); 384 - 0.4 µM Mirin[16] (MedChemExpress LLC, NJ, USA); 640 - 0.6 µM C5[17] (MedChemExpress LLC, NJ, USA); 640 - 0.6 µM AZD-6738[18] (Axon Medchem LLC, VA, USA); 80 - 0.04 µM PF-477736[19] (Biomol GmbH, Hamburg, Germany); 35.2 - 0.02 µM L-Dopa[20] (MedChemExpress LLC, NJ, USA); 0.11 – 108.8 nM XL413[21] (MedChemExpress LLC, NJ, USA9; 1000 – 0.49 µM Olaparib[22] (MedChemExpress LLC, NJ, USA); 64 – 0.06 µM Doxorubicin (Pharmacy of the University Hospital Ulm)[23]; 431 – 0.42 µM Carboplatin (Pharmacy of the University Hospital Ulm)[24]. For combination treatments, the IC20 values of JH-RE-06 (1.3 µM), PF-477736 (1.5 µM), B02 (174.6 µM), obtained from the mean of all three cell lines in both culture conditions in the respective mono-treatments, were used in combination with 1000 – 0.49 µM Olaparib. The IC20 concentration was held constant during Olaparib titration. To assess multidrug resistance (MDR) the cells were preincubated with 50 µM Verapamil[25] for 1 h prior to combination treatment with 1000 – 0.49 µM Olaparib and constant Verapamil concentration of 50 µM.

Supplementary References

1 Dai X, Cheng H, Bai Z, Li J. Breast Cancer Cell Line Classification and Its Relevance with Breast Tumor Subtyping. *J Cancer* 2017; **8**: 3131–3141.

2 Cellosaurus MDA-MB-453 (CVCL_0418). https://www.cellosaurus.org/CVCL_0418 (accessed 5 May2026).

3 Cellosaurus BT-474 (CVCL_0179). https://www.cellosaurus.org/CVCL_0179 (accessed 5 May2026).

4 Cellosaurus T-47D (CVCL_0553). https://www.cellosaurus.org/CVCL_0553 (accessed 5 May2026).

5 Cellosaurus MCF-7 (CVCL_0031). https://www.cellosaurus.org/CVCL_0031 (accessed 5 May2026).

6 Cellosaurus MDA-MB-134-VI (CVCL_0617). https://www.cellosaurus.org/CVCL_0617 (accessed 5 May2026).

7 Koch C, Kuske A, Joosse SA, Yigit G, Sflomos G, Thaler S *et al.* Characterization of circulating breast cancer cells with tumorigenic and metastatic capacity. *EMBO Mol Med* 2020; **12**: e11908.

8 Cellosaurus MDA-MB-231 (CVCL_0062). https://www.cellosaurus.org/CVCL_0062 (accessed 5 May2026).

9 Cellosaurus Hs 578T (CVCL_0332). https://www.cellosaurus.org/CVCL_0332 (accessed 5 May2026).

10 Cellosaurus MDA-MB-468 (CVCL_0419). https://www.cellosaurus.org/CVCL_0419 (accessed 5 May2026).

11 Cellosaurus MDA-MB-436 (CVCL_0623). https://www.cellosaurus.org/CVCL_0623 (accessed 5 May2026).

12 Zafar MK, Maddukuri L, Ketkar A, Penthala NR, Reed MR, Eddy S *et al.* A Small-Molecule Inhibitor of Human DNA Polymerase η Potentiates the Effects of Cisplatin in Tumor Cells. *Biochemistry* 2018; **57**: 1262–1273.

13 Patel SM, Dash RC, Hadden MK. Translesion synthesis inhibitors as a new class of cancer chemotherapeutics. *Expert Opin Investig Drugs* 2021; **30**: 13–24.

14 Wojtaszek JL, Chatterjee N, Najeeb J, Ramos A, Lee M, Bian K *et al.* A Small Molecule Targeting Mutagenic Translesion Synthesis Improves Chemotherapy. *Cell* 2019; **178**: 152-159.e11.

15 Shkundina IS, Gall AA, Dick A, Cocklin S, Mazin A V. New rad51 inhibitors to target homologous recombination in human cells. *Genes (Basel)* 2021; **12**. doi:10.3390/genes12060920.

16 Dupré A, Boyer-Chatenet L, Sattler RM, Modi AP, Lee J-H, Nicolette ML *et al.* A forward chemical genetic screen reveals an inhibitor of the Mre11–Rad50–Nbs1 complex. *Nat Chem Biol* 2008; **4**: 119–125.

17 Liu W, Zhou M, Li Z, Li H, Polaczek P, Dai H *et al.* A Selective Small Molecule DNA2 Inhibitor for Sensitization of Human Cancer Cells to Chemotherapy. *EBioMedicine* 2016; **6**: 73–86.

18 Chiappa M, Guffanti F, Anselmi M, Lupi M, Panini N, Wiesmüller L *et al.* Combinations of ATR, Chk1 and Wee1 Inhibitors with Olaparib Are Active in Olaparib Resistant Brca1 Proficient and Deficient Murine Ovarian Cells. *Cancers (Basel)* 2022; **14**. doi:10.3390/cancers14071807.

19 Mann J, Niedermayer K, Krautstrunk J, Abbey L, Wiesmüller L, Piekorz RP *et al.* Combined inhibition of RAD51 and CHK1 causes synergistic toxicity in cisplatin resistant cancer cells by triggering replication fork collapse. *Int J Cancer* 2025; **156**: 389–402.

20 Chandramouly G, McDevitt S, Sullivan K, Kent T, Luz A, Glickman JF *et al.* Small-Molecule Disruption of RAD52 Rings as a Mechanism for Precision Medicine in BRCA-Deficient Cancers. *Chem Biol* 2015; **22**: 1491–1504.

21 Koltun ES, Tsuhako AL, Brown DS, Aay N, Arcalas A, Chan V *et al.* Discovery of XL413, a potent and selective CDC7 inhibitor. *Bioorg Med Chem Lett* 2012; **22**: 3727–3731.

22 Malka MM, Eberle J, Niedermayer K, Zlotos DP, Wiesmüller L. Dual parp and rad51 inhibitory drug conjugates show synergistic and selective effects on breast cancer cells. *Biomolecules* 2021; **11**. doi:10.3390/biom11070981.

23 Eberle J, Wiehe RS, Gole B, Mattis LJ, Palmer A, Ständker L *et al.* A Fibrinogen Alpha Fragment Mitigates Chemotherapy-Induced MLL Rearrangements. *Front Oncol* 2021; **11**. doi:10.3389/fonc.2021.689063.

24 Deniz M, Romashova T, Kostezka S, Faul A, Gundelach T, Moreno-Villanueva M *et al.* Increased single-strand annealing rather than non-homologous end-joining predicts hereditary ovarian carcinoma. *Oncotarget* 2017; **8**: 98660–98676.

25 Biegała Ł, Gajek A, Marczak A, Rogalska A. Olaparib-Resistant BRCA2MUT Ovarian Cancer Cells with Restored BRCA2 Abrogate Olaparib-Induced DNA Damage and G2/M Arrest Controlled by the ATR/CHK1 Pathway for Survival. *Cells* 2023; **12**. doi:10.3390/cells12071038.
