# Supplementary material for: Targeting common denominators of DNA damage tolerance mechanisms as therapeutic strategy to cope with dynamic phenotypic changes in triple-negative breast cancer cells
Source: Cell Death Discov. 2026 Oct 6;12:405. doi: 10.1038/s41420-026-03377-4 (PMC13642338; doi:10.1038/s41420-026-03377-4)
Supplement: Supplementary file 4 [file 41420_2026_3377_MOESM4_ESM.pdf]

Source Data ad Supplementary Figures 1b/2c:
DMSO and Olaparib treatment
(Uncropped Western Blots)

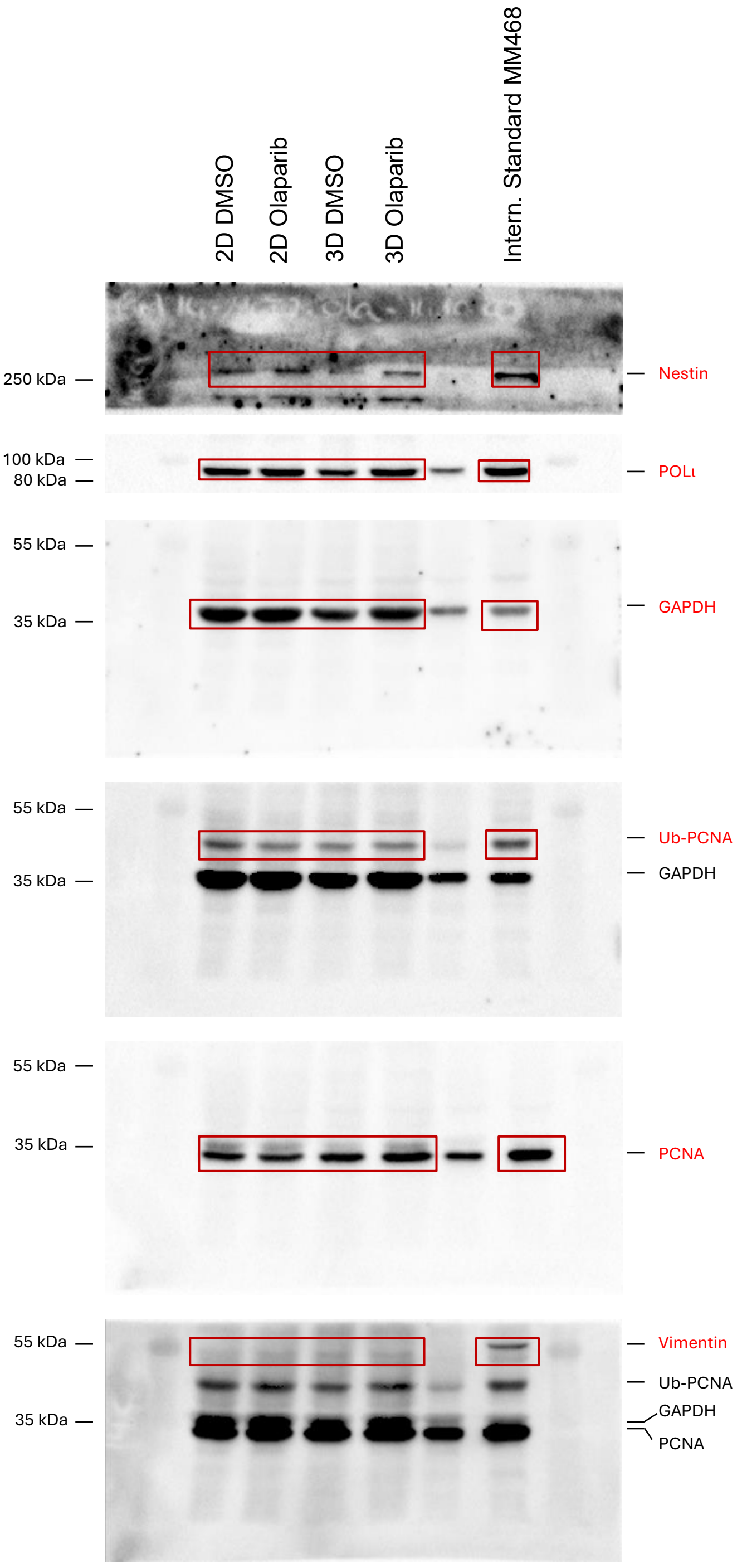

MCF-7

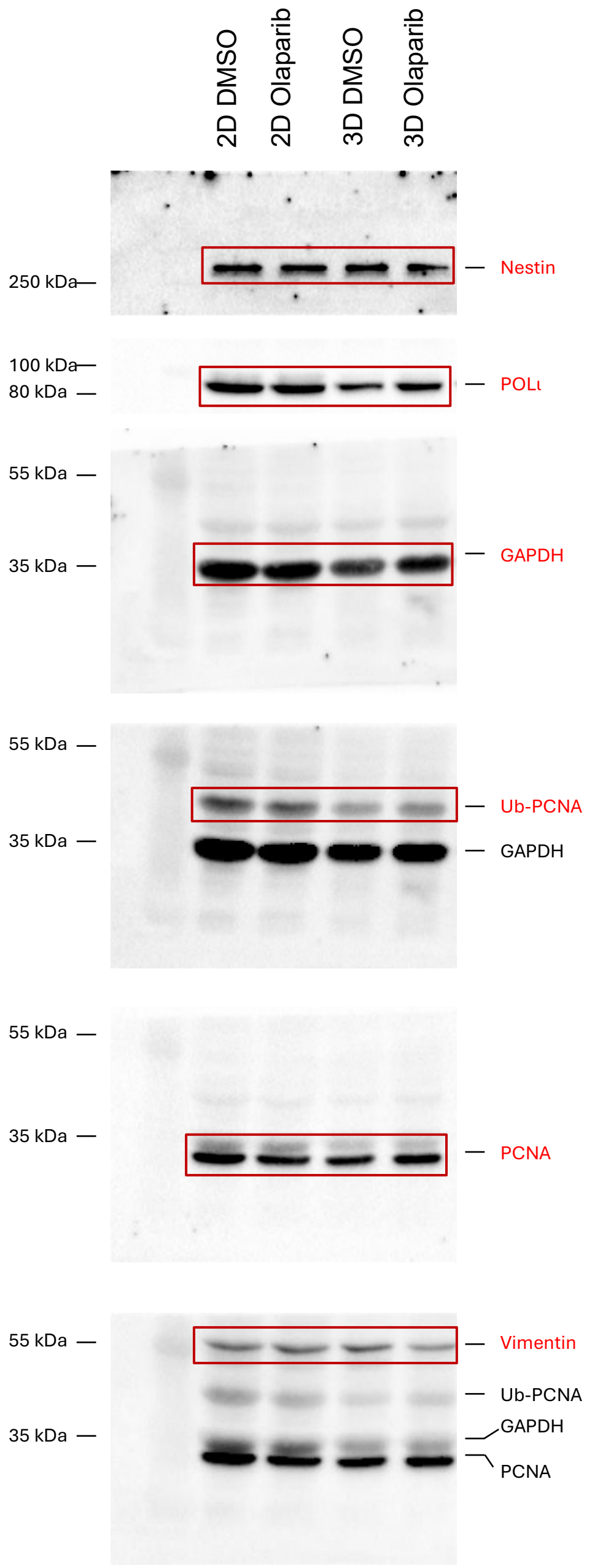

MDA-MB-468

Shown are the uncropped western blot images of DMSO and Olaparib treated samples from Supplementary Figures 1b and 2c. The red boxes indicate where the images were cropped. The proteins of the cropped bands are indicated in red, other proteins immunodetected on the same blot are indicated in black. The same GAPDH blots were shown in both Supplementary Figures 1b and 2c as loading controls for the respective cell lines, since all shown proteins were detected on the same blot.

Source Data ad Supplementary Figures 1b/2c: H_2O, Carboplatin and Doxorubicin treatment (Uncropped Western Blots)

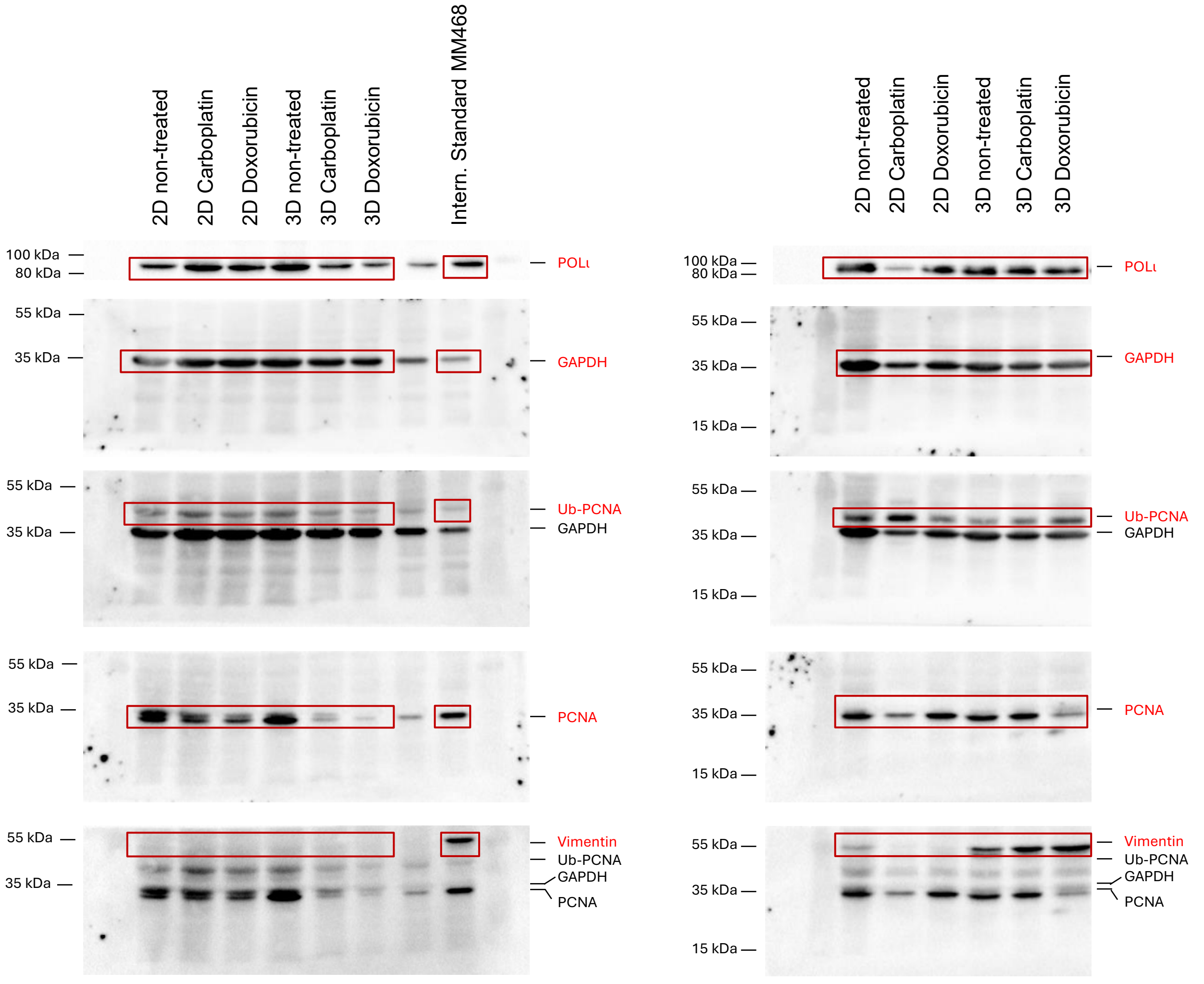

MCF-7

MDA-MB-468

Shown are the uncropped western blot images of non-treated (H_2O), Carboplatin and Doxorubicin treated samples from Supplementary Figures 1b and 2c. The red boxes indicate where the images were cropped. The proteins of the cropped bands are indicated in red, other proteins on the same blot are indicated in black. The same GAPDH blots were shown in both Supplementary Figures 1b and 2c as loading controls for the respective cell lines, since all shown proteins were detected on the same blot.

Source Data ad Supplementary Figure 7c: DNA damage (Uncropped Western Blot)

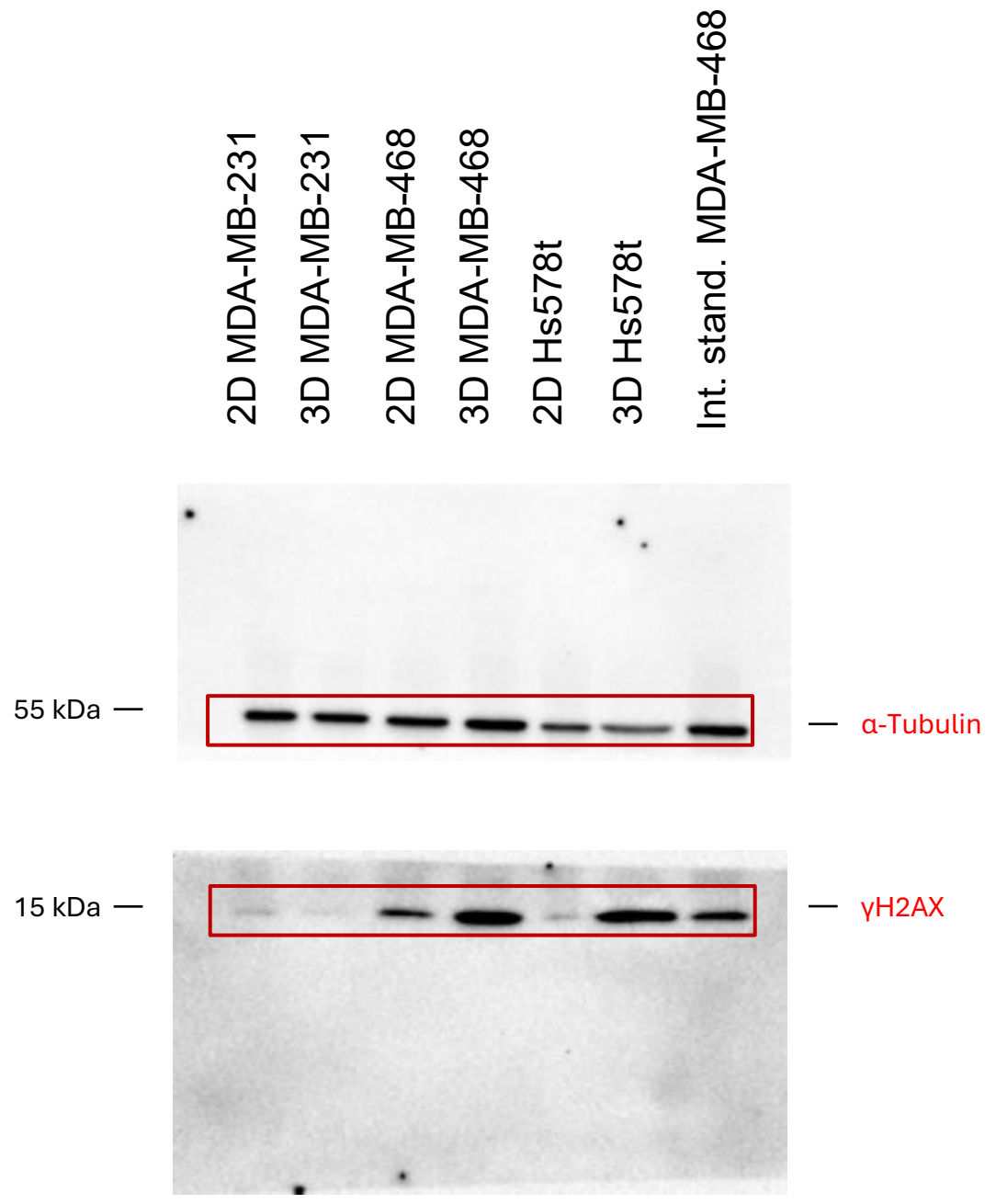

Shown are the uncropped western blot images of non-treated samples from Supplementary Figures 7c. The red boxes indicate where the images were cropped. The proteins of the cropped bands are indicated in red.
